# Halophilic archaea produce wax esters and use an alternative fatty acyl-coenzyme A reductase for precursor synthesis

**DOI:** 10.1093/ismejo/wraf035

**Published:** 2025-02-24

**Authors:** Vincent Grossi, Philippe Cuny, Cécile Militon, Jerzy Witwinowski, Balkis Eddhif, Léa Sylvi, Mireille Nowakowski, Artemis Kosta, Ingrid Antheaume, Johan Cornil, Sarah Dubrac, Julia Kende, Simonetta Gribaldo, Guillaume Borrel

**Affiliations:** Aix Marseille Univ, Université de Toulon, CNRS, IRD, Mediterranean Institute of Oceanography (MIO), Marseille, France; Aix Marseille Univ, Université de Toulon, CNRS, IRD, Mediterranean Institute of Oceanography (MIO), Marseille, France; Aix Marseille Univ, Université de Toulon, CNRS, IRD, Mediterranean Institute of Oceanography (MIO), Marseille, France; Institut Pasteur, Université Paris Cité, UMR CNRS 6047, Unit Evolutionary Biology of the Microbial Cell, Paris, France; UCBL, CNRS, ENS, Laboratoire de Géologie de Lyon, Terre, Planètes, Environnement (LGL-TPE), Univ Lyon, Villeurbanne, France; Aix Marseille Univ, Université de Toulon, CNRS, IRD, Mediterranean Institute of Oceanography (MIO), Marseille, France; Institut Pasteur, Université Paris Cité, C2RT-Production and Purification of Recombinant Proteins Technological Platform, Paris, France; Institut de Microbiologie de la Méditerranée, IMM, FR3479, Campus CNRS, Marseille, France; UCBL, CNRS, ENS, Laboratoire de Géologie de Lyon, Terre, Planètes, Environnement (LGL-TPE), Univ Lyon, Villeurbanne, France; Institut Pasteur, Université Paris Cité, UMR CNRS 3523, Chem4Life, Unit Chemistry of Biomolecules, Paris, France; Institut Pasteur, Université Paris Cité, UMR CNRS 6047, Unit Stress Adaptation and Metabolism in enterobacteria, Paris, France; Institut Pasteur, Université Paris Cité, Bioinformatics and Biostatistics Hub, Paris, France; Institut Pasteur, Université Paris Cité, UMR CNRS 6047, Unit Evolutionary Biology of the Microbial Cell, Paris, France; Institut Pasteur, Université Paris Cité, UMR CNRS 6047, Unit Evolutionary Biology of the Microbial Cell, Paris, France

**Keywords:** fatty acyl-CoA reductase, halophilic archaea, neutral lipids, (poly)extreme environments, wax ester biosynthesis

## Abstract

Wax esters (WE) are fatty acid-based neutral lipids thought to be restricted to bacteria and eukaryotes, playing a key role in the functioning and maintenance of cells, especially under adverse conditions. Here, we show that several halophilic archaea (*Halobacteriales*) carry a homolog of the bacterial wax synthase gene. WE synthesis and accumulation are demonstrated in one of these (poly)extremophilic archaea, *Natronomonas pharaonis*, during growth on long-chain fatty acids. Our bioinformatic analysis also shows that the synthesis of fatty alcohols required for WE synthesis could be performed by an enzyme evolutionarily related to Class-I 3-hydroxy-3-methylglutaryl-coenzyme A (HMG-CoA) reductase (HMGR, classically involved in the isoprenoid biosynthesis pathway). Using heterologous expression and enzymatic assays, we show that this HMGR homolog, which we named FcrA (for fatty acyl-CoA reductase), reduces fatty acyl-CoA to fatty alcohol but cannot reduce HMG-CoA to mevalonate, contrasting with the canonical HMGR. The conservation of HMGR catalytic residues in FcrA suggests that the two enzymes have a similar catalytic mechanism, whereas an elongated substrate-binding pocket and distinct residues may explain FcrA's selectivity for fatty acyl-CoA. In addition to archaea, FcrA is present in a wide range of bacteria, including ~25% of those predicted to produce WEs, and accounts for a large proportion of the fatty acyl-CoA reductases found in various environments. Challenging the long-held paradigm that archaea cannot biosynthesize fatty acid-based neutral lipids *de novo*, this study lays the foundation for further physiological, ecological, and biotechnological investigations of neutral lipid production by systems markedly different from those of eukaryotes and bacteria.

## Introduction

**Figure 1:**
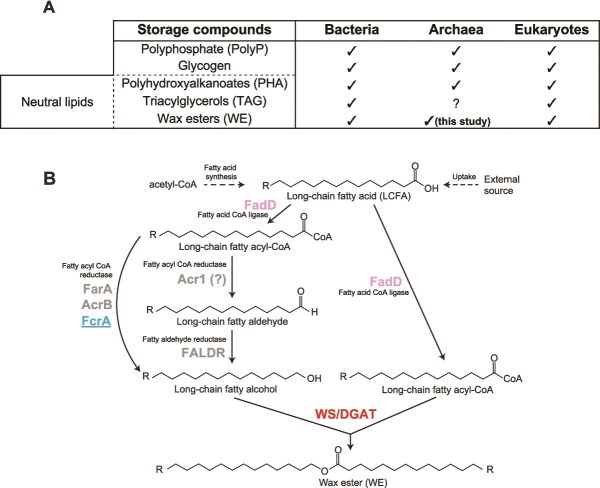
Distribution of storage compounds and WE biosynthetic pathways in the three domains of life. (A) Storage compounds described so far in eukaryotes and prokaryotes. The production of WE by archaea is described in this study. (B) Biosynthetic pathways to WE production. A long-chain fatty acid is activated to fatty acyl-coenzyme A (acyl-CoA) by a fatty acyl-CoA ligase (FadD). Part of the fatty acyl-CoA is reduced to the corresponding fatty alcohol by different types of fatty acyl-CoA reductases. FcrA is the fatty acyl-CoA reductase characterized in this study. WE synthesis is then catalyzed by the WE synthase WS/DGAT, which uses a fatty acyl-CoA as the activated substrate and a fatty alcohol as the acceptor molecule.

The storage of energy compounds in the form of intracellular inclusions is a fundamental metabolic process in eukaryotes and prokaryotes, playing a key role in cell physiology and maintenance [1–3]. Various classes of storage compounds have been described, including polyphosphate (PolyP), glycogen, and three classes of neutral lipids: polyhydroxyalkanoates (PHA), triacylglycerols (TAG), and wax esters (WE) (Fig. 1A).

The production and intracellular accumulation of neutral lipids have been extensively documented in unicellular microorganisms such as yeasts, microalgae, bacteria, and archaea [1, 4]. Under unbalanced growth conditions, such as nitrogen-limitation, some microorganisms can accumulate up to a third of their cell dry weight as neutral lipids [5, 6]. This accumulation of storage lipids results in the formation of intracellular fat bodies (called lipid inclusions or lipid droplets), which can contribute to cell maintenance under adverse conditions (source of energy and carbon during starvation), cell division, stress response, or virulence in pathogenic bacteria [3, 6, 7]. Due to various ecological, environmental, and biotechnological interests, the biochemical and genetic basis of WE and TAG biosynthesis in bacteria has been intensively documented over the last two decades (e.g. [3, 6–9]).

Multiple steps are involved in the synthesis of WE and TAG in bacteria, the final one being carried out by a bifunctional enzyme known as WE synthase/acyl-coenzyme A:diacylglycerol acyltransferase (WS/DGAT). Different WS/DGAT can act as both WE synthase (acyl-CoA:fatty alcohol acyltransferase, WS) and DGAT, or perform only one of these functions [6]. Initially discovered in *Acinetobacter baylyi* ADP1 (formerly *Acinetobacter calcoaceticus*; [10]), WS/DGAT homologs have subsequently been reported from numerous bacteria [6, 11] and environmental samples [12]. A bioinformatics study of 8282 bacterial genomes showed that 673 species, mainly *Proteobacteria* and *Actinobacteria*, harbor one or several copies of the *WS/DGAT* gene [9]. WS/DGAT is generally promiscuous and accepts a wide variety of acyl-coenzyme A (acyl-CoA) substrates as acyl donor, and fatty alcohols or diacylglycerols as acyl acceptors for WE or TAG formation, respectively [10, 13]. The ability to produce WE and/or TAG is strain-dependent and can be influenced by the nature of the growth substrate and metabolic intermediates [3, 14, 15]. The fatty acyl-CoA and fatty alcohol used by the WS/DGAT to produce WE (Fig. 1B) are derived from long-chain fatty acids (LCFA) taken from the environment or produced by the cell. LCFA are activated to fatty acyl-CoA by a fatty acyl-CoA ligase (FadD; Fig. 1B). Two different enzymes, FarA and AcrB, which share <30% sequence identity, can then produce directly fatty alcohol via a NADPH-based four-electron reduction of fatty acyl-CoA ([16–18]; Fig. 1B). Alternatively, this step can be performed sequentially in two steps involving two enzymes, Acr1 for the reduction of fatty acyl-CoA to fatty aldehyde and an unknown fatty aldehyde reductase (FALDR) for the reduction of fatty aldehyde to fatty alcohol ([19]; Fig. 1B).

Although a WS/DGAT homolog has been reported once in the haloalkaliphilic archaeon *Natronomonas pharaonis* [8], current data suggest that archaea do not have the ability to produce WE or TAG [1, 3, 4]. This is consistent with a bioinformatics study of archaeal energy metabolism based on over 400 archaeal reference genomes, which reported the absence of WS/DGAT homologs as well as TAG and WE metabolic pathways in archaea, in contrast to the presence of pathways for the synthesis of PolyP, PHA, and glycogen [20]. To date, the production of WE or TAG by an archaeal strain has never been demonstrated [3].

In this study, we questioned the presence of these neutral fatty acyl-based lipids in archaea. Based on bioinformatics investigations, strain cultures, lipidomic analyses, experimental heterologous gene expression, and enzymatic assays, we demonstrate that members of the *Halobacteriales* are capable of producing WE by involving a newly characterized enzyme (Fig. 1) that has a wide environmental and taxonomic distribution, extending beyond extremophilic archaea.

## Materials and methods

### Archaeal strains and culture conditions

*Natronomonas pharaonis* type strain Gabara [21] and *Natronomonas moolapensis* CSW 8.8.11 [22] were purchased from the German Collection of Microorganisms and Cell Cultures GmbH (DSMZ; strains DSM2160 and DSM371, respectively) and grown in medium DSM 371 (which we refer to as N-rich; Supplementary methods). A nitrogen-limited (N-limited) medium was prepared by excluding casamino acids and Na_2_-glutamate and lowering the concentrations of NH_4_Cl and yeast extract to 0.05 and 0.25 g/l, respectively, compared with DSM 371 medium (see Supplementary methods).

For biomass production, the two species of *Natronomonas* were grown in medium 371 at 37°C and 90 rpm. Cells were collected by centrifugation (10 min, 4000 g) at the end of the exponential growth phase and washed three times with N-limited medium. The washed cells were then inoculated (4.87E + 09 ± 1.62E + 09 cells per 50 ml) in N-limited and N-rich (medium 371) media supplemented with either LCFA (palmitic acid [C_16:0_] or oleic acid [C_18:1_] at a concentration of 2 mg ml^−1^, or a mixture of both, each at a concentration of 1 mg ml^−1^) or soluble substrates (SS; sodium acetate and pyruvate, 2 mg ml^−1^ of each), and incubated for 10–20 days. The potential production of WE and TAG by the two *Natronomonas* species was investigated in different individual cultures grown under the four above-mentioned growing conditions (N-rich or N-limited and LCFA or SS). Abiotic controls consisting of growth medium inoculated with thermally inactivated cells (120°C for 20 min) were incubated in parallel.

The kinetics of WE production by *N. pharaonis* strain DSM 2160 was carried out in parallel with cell count. Individual 50 ml cultures were incubated for 1, 2, 5, 7, and 10 days under N-limited/LCFA conditions. Replicate cultures (six per sampling time) were stopped immediately after inoculation (Day 0) and after each incubation period. For cell density measurement, a subsample (3 ml) of three of the six replicate cultures was filtered through a cellulose acetate membrane filter (0.2 μm), which was immediately stored at −20°C. The cell density was estimated by quantitative PCR (Supplementary methods). To optimize WE recovery, WE were quantified in the other three replicate cultures, which were stored directly at −20°C. Additional cultures grown under the other conditions considered (N-limited/SS, N-rich/LCFA, N-rich/SS) and abiotic controls were stopped after Day 10. Cultures grown under N-limited/LCFA and N-limited/SS conditions were used for electron microscopy observations (Supplementary methods).

### Lipid extraction and separation

Neutral lipids were extracted from each individual culture by ultrasonication using dichloromethane (DCM, x3). After each extraction, the mixture was centrifuged (5 min at 4200 rpm) and the DCM phase was pipetted out. The combined extracts were dried over anhydrous sodium sulfate, filtered over pre-cleaned cotton, concentrated by rotary evaporation, and evaporated to dryness under N_2_ flow. The total lipid extract was then chromatographed over a silica gel column (Merck silica gel 60), and fractions of increasing polarity were eluted using *n*-heptane (Hept), Hept-DCM (1:1, v/v), Hept-DCM (1:3, v/v), and Hept-Ethyl Acetate (4:1, v/v). The last three fractions, potentially containing WE, TAG, and free alcohols respectively, were concentrated before analysis by gas chromatography (GC) and GC–mass spectrometry (GC–MS) (Supplementary methods). Alcohols were analyzed as silylated derivatives obtained using *N*,*O*-Bis(trimethylsilyl)trifluoroacetamide (BSTFA) and pyridine (1:2 v/v, 45 min at 50°C).

### Screening for wax ester synthesis pathway in archaeal genomes

An archaeal database was built by downloading all 5679 archaeal genomes available in GenBank in August 2022. WS/DGAT homologs were searched in this database with BLASTp, using the reference WS/DGAT sequence of *A. baylyi* ADP1 (WP_004922247.1) as query, and using hmmsearch with the profile TIGR02946. Collected hits were considered to correspond to WS/DGAT on the basis of their alignment with other bacterial WS/DGAT and of the fully or largely conserved HHXXXDG motif. In addition, other enzymes known to be involved in the WE (FadD, FarA, AcrB, and Acr1) and TAG (GlpA, GlpK, GpsA, UgpA, UgpB, UgpE, GPAT, AGPAT, and PAP) synthesis pathways, and those of the β-oxidation pathway (FadA, FadB-Nter/C-ter, FadE, EtfA, and EtfB) were searched in a selection of 64 *Halobacteria* genomes using hmmsearch.

### Screening for fatty acyl-CoA reductase in *Halobacteriales*

A database of 44 complete or nearly complete *Halobacteriales* genomes covering the phylogenetic diversity of the lineage was assembled. Thirteen of them coded for a WS/DGAT. From this database, 40 464 protein families were defined using Silix, with a 45% identity and 80% coverage cut-off [23]. The distribution pattern of the presence/absence of each protein family was correlated (Pearson correlation) with the distribution of WS/DGAT. Protein families with the best Pearson correlation coefficient were selected for further analysis.

### Phylogenetic analyses

All maximum likelihood trees were built with IQ-TREE 2.0.6 with the TESTNEW option to define the best model [24]. Tree visualization, coloring, and protein presence/absence mapping were done with ITOL [25]. A tree of *Halobacteriales*, including the 26 *Halobacteriales* genomes coding for WS/DGAT, was built using the Phylosift dataset [26] and five additional markers as described in [27]. For the WS/DGAT tree, bacterial sequences were searched in a taxonomically equilibrated database of 1093 bacteria (Bac-1093) covering all major phyla [28]. They were pooled with the 30 *Halobacteriales* sequences and aligned with MAFFT L-INS-I. WS/DGAT maximum-likelihood tree was built with the LG + F + R5 model. For the HMGR/FcrA tree, an ABE database covering the three domains of life was assembled from the Bac-1093 database, as well as 127 archaea and 86 eukaryotes genomes covering major lineages in these domains. HMGR/FcrA was search in this database using HMMSEARCH with the PF00368 profile. HMGR/FcrA maximum-likelihood tree was built with the LG + R9 model.

### Taxonomic and environmental distribution of wax ester synthase/acyl-CoA:diacylglycerol acyltransferase, fatty acyl-CoA reductase, FarA, and AcrB

WS/DGAT and the three fatty acyl-CoA reductase (FcrA, FarA, and AcrB) were searched in 52 515 genomes from the of Earth's microbiomes catalog covering a large range of environments [29]. Search was carried out with HMMSEARCH using TIGR02946, PLN02503, and PRK07201 profiles for WS/DGAT, FarA, and AcrB, respectively, and a manually curated profile for FcrA. Taxonomic distribution was determined based on GTDB taxonomy obtained with GTDBTk 2.1.1 [30]. Environmental categories were delineated from sample descriptions provided in the original publication of [29].

### Structural models of fatty acyl-CoA reductase and 3-hydroxy-3-methylglutaryl-CoA reductase

Structures of 59 FcrA and 46 Class-I HMGR identified from the ABE database were predicted as homotetramer using AlphaFold 3 [31]. Because fatty-acyl CoA ligands were not available on the AlphaFold 3 server, we used palmitic acid and myristic acid to infer the position of the fatty-acyl CoA carbon chain in FcrA. Four to eight palmitic acid (or myristic acid) molecules and four NADPH were included in FcrA models. Mapping of amino acid conservation on *N. pharaonis* FcrA structure was performed with Consurf 2016 [32]. Images of structures were generated in PyMOL (https://pymol.org/). Mapping of amino acid conservation and secondary structure of *N. pharaonis* FcrA were generated with Espript 3.0 [33].

### Expression vector construction

All plasmids and primers used are listed in Supplementary Tables S1 and S2, respectively. Codon sequences of *WS/DGAT* from *A. baylyi* ADP1 (WP_004922247.1) and *fcrA* (CUS01882.1) from “Ca. Promineifilum breve” (referred to as *Ab-WS/DGAT* and *Pb-fcrA*, respectively) were optimized for *Escherichia coli* using a Novoprolabs tool (https://www.novoprolabs.com/tools/codon-optimization). Adapted codon sequences were synthetized *de novo* by Eurofins as an artificial operon, where FcrA was separated from WS/DGAT by a short linker containing a strong RBS described in [34].

To construct a vector expressing both FcrA and WS/DGAT, the synthetic genetic construct was amplified with JW381/JW382 primer pair and cloned into pET22b between NdeI and XhoI restriction sites, yielding pJW105 plasmid (adding a C-terminal 6His tag to the WS/DGAT protein sequence). The expression of tagged WS/DGAT was confirmed by a western blot (Supplementary Fig. S1). To construct a vector expressing only FcrA, the *WS/DGAT* was eliminated from the pJW105 plasmid by EcoRI/XhoI restriction digestion, followed by generating blunt ends with the Klenow fragment (Thermo Fisher Scientific) and self-circularization with T4 DNA ligase (Thermo Fisher Scientific), yielding the pJW111 plasmid. To construct a vector expressing only WS/DGAT, the corresponding gene has been amplified from the pJW105 plasmid with JW399/JW382 primer pair and cloned into pET22b between NdeI and XhoI restriction sites, yielding the pJW114 plasmid (adding a C-terminal 6His tag to the WS/DGAT sequence). The integrity of all inserts and vectors was verified by Sanger sequencing.

### Heterologous gene expression in *Escherichia coli*

Pb-FcrA and Ab-WS/DGAT were expressed in *E. coli* BL21(DE3) strains containing appropriate plasmids were grown in auto-induction medium containing 1% tryptone, 0.5% yeast extract, 42 mM Na_2_HPO_4_, 22 mM KH_2_PO_4_, 86 mM NaCl, 2 mM MgSO_4_, 0.2% (v/v) trace element solution [35], 0.05% glucose, 0.2% α-lactose, and either 0.5% palmitic acid or 0.5% glycerol. A transformed colony on lysogeny broth (LB) plate was transferred in 5 ml of LB medium with the appropriate antibiotic and grown overnight at 37°C. One milliliter of this pre-culture was used to inoculate 50 ml of fresh autoinduction medium with antibiotic. The cultures were incubated at 37°C (250 rpm) for 30 h before cells were harvested for lipid analysis.

### Enzymatic assays

Pb-FcrA was expressed and purified as presented in Supplementary methods. All enzymatic assays were performed in the final volume of 1 ml. The reaction mix contained potassium phosphate buffer 0.5 M (pH 7.5), potassium chloride 20 mM, dithiothreitol 10 mM, NADPH or NADH 300 μM, FcrA 36 nM, and the tested substrate (fatty acyl-CoA [C_4:0_, C_8:0_, C_12:0_, C_14:0_, C_16:0_, C_18:1_], cis-11-hexadecenal or HMG-CoA) at 200 μM. When needed, activated simvastatin (see Supplementary methods) was added at 10 μM. Enzymatic assays were performed in the Ruskinn Concept 500 anaerobic chamber filled with a N_2_/CO_2_/H_2_ 90:5:5 (v/v/v) gas mixture heated to 37°C, using water and liquid solutions pre-incubated in anoxia for 24 h. Reactions were launched by the addition of the substrate (fatty acyl-CoA or HMG-CoA). The decrease of absorbance at 340 nm (NAD(P)H maximum) was used to measure NAD(P)H oxidation. Absorbance from a reaction mix without fatty acyl-CoA was used as comparison after 1 h of incubation.

## Results and discussion

### Occurrence of wax ester biosynthesis pathway in archaea

**Figure 2:**
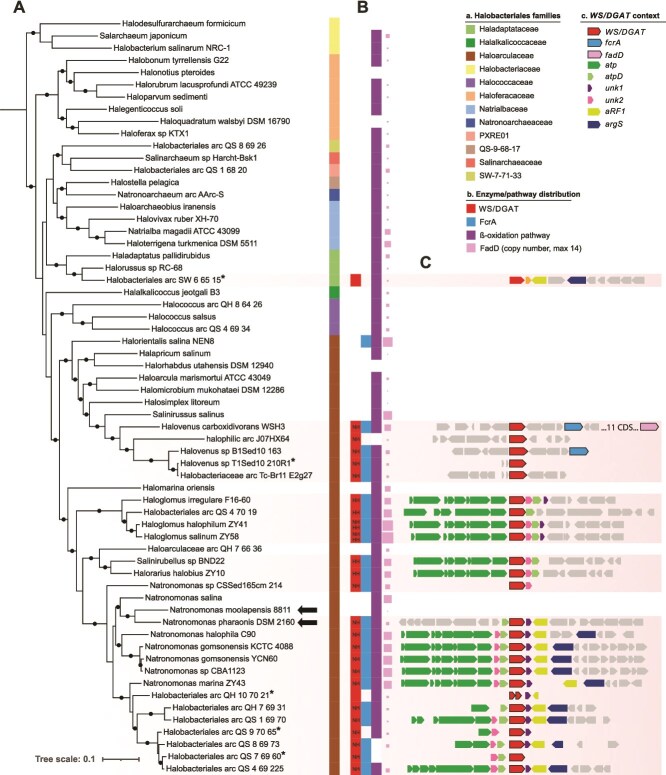
Phylogenic distribution and genomic context of WS/DGAT in *Halobacteriales* taxa. (A) Maximum-likelihood tree (LG + F + R10) of *Halobacteriales* based on concatenation of 38 conserved phylogenetic markers (10 562 amino acid positions) and 64 genomes/MAGs. Node supports refer to ultrafast bootstrap values, with only values above 0.9 shown. The tree was rooted using Hikarchaea as outgroup (not shown). Arrows indicate the two *Halobacteriales* species used in this study, *N. pharaonis* DSM 2160 coding for WS/DGAT and the closest related species *N. moolapensis* 8811, not coding for WS/DGAT. MAGs of taxa marked with an asterisk (*) are estimated <70% complete. (B) Distribution of *WS/DGAT*, of the novel fatty acyl-CoA reductase (*fcrA*) identified in this study, of the β-oxidation pathway and *fadD* copy number, in the 64 *Halobacteriales*. “NH” and “HH” indicate the presence of an NHXXXDG and HHXXXDG motif, respectively, in WS/DGAT. Two taxa have two WS/DGAT, one with an NHXXXDG motif and the other with an HHXXXDG motif. (C) Genomic context of *WS/DGAT* in *Halobacteriales*. Genes in gray are not conserved around *WS/DGAT* in more than nine genomes, with the exception of *fcrA* and *fadD*. The *fcrA* gene is not commonly close to *WS/DGAT*, but is almost exclusively present in *Halobacteriales* coding for *WS/DGAT*.

We examined the presence of genes coding for WS/DGAT in 5679 archaeal genomes and detected its presence in 26 halophilic archaea belonging to the order *Halobacteriales* (Fig. 2), some having two copies of the gene. The *WS/DGAT* gene was also detected in a metagenome-assembled genome (MAG) of ANME-2a (GCA_014237125.1). However, its nucleotide sequence is 100% identical to that of a gene present in a bacterial MAG (*Desulfobacteraceae*) reconstructed from the same metagenome, indicating cross-contamination during sequence processing. Most *Halobacteriales* encoding WS/DGAT belong to the family *Haloarculaceae* and include *Natronomonas* spp. (including *N. pharaonis*), the recently isolated *Haloglomus* spp., *Halorarius* sp., and *Halovenus* spp. [36–38], as well as MAG from salt crusts of the Atacama Desert [39] (Fig. 2). This narrow phyletic distribution in Archaea indicates that it was acquired through horizontal gene transfer, likely within *Halobacteriales*. The closest sequence to *Halobacteriales* in the WS/DGAT phylogeny belongs to a halophilic bacterium, *Persicimonas caeni* (Supplementary Fig. S2), supporting a transfer from bacteria sharing the same environment.

Archaeal and bacterial WS/DGAT show a high level of sequence conservation (Supplementary Fig. S3). However, the HHXXXDG motif present in the catalytic site of bacterial WS/DGAT, is not fully conserved in 22 of the 30 archaeal WS/DGAT, including *N. pharaonis*. In these archaea, His132 is replaced by an Asn, leading to a NHXXXDG motif (Fig. 2B and Supplementary Figs S2 and S3). In bacterial WS/DGAT, His132 has a key structural role, whereas His133 is directly responsible for WS/DGAT catalytic activity [40]. The critical role of these histidines was also revealed by a drop in *A. baylyi* WS/DGAT activity when they are replaced by other amino acids [41].

Other enzymes involved in the WE and TAG synthesis pathways were sought in *Halobacteriales* genomes coding for WS/DGAT*.* Whereas all these archaea encode several FadD homologs involved in the activation of LCFA (Figs 1B and 2B and Supplementary Fig. S4A), none of them have FarA and AcrB, involved in the reduction of fatty acyl-CoA to the corresponding fatty alcohols in the WE pathway (Fig. 1B). Distant homologs of Acr1, involved in the reduction of fatty acyl-CoA to the corresponding fatty aldehyde (Fig. 1B), were identified in all *Halobacteriales*. However, Acr1 belongs to a very large protein family (short-chain dehydrogenase/reductase family, SDR) with many different functions [42], suggesting that these distant homologs have a completely different function than fatty acyl-CoA reduction. In addition, except for WS/DGAT, most of the enzymes involved in TAG synthesis pathway are also absent from *Halobacteriales* (Supplementary Fig. S4).

At first sight, the presence of an unusual WS/DGAT NHXXXDG motif and the potential incompleteness of the associated pathways in *Halobacteriales* raised questions about their ability to synthesize WE and/or TAG.

### Production of wax esters in *Natronomonas pharaonis*

**Figure 3:**
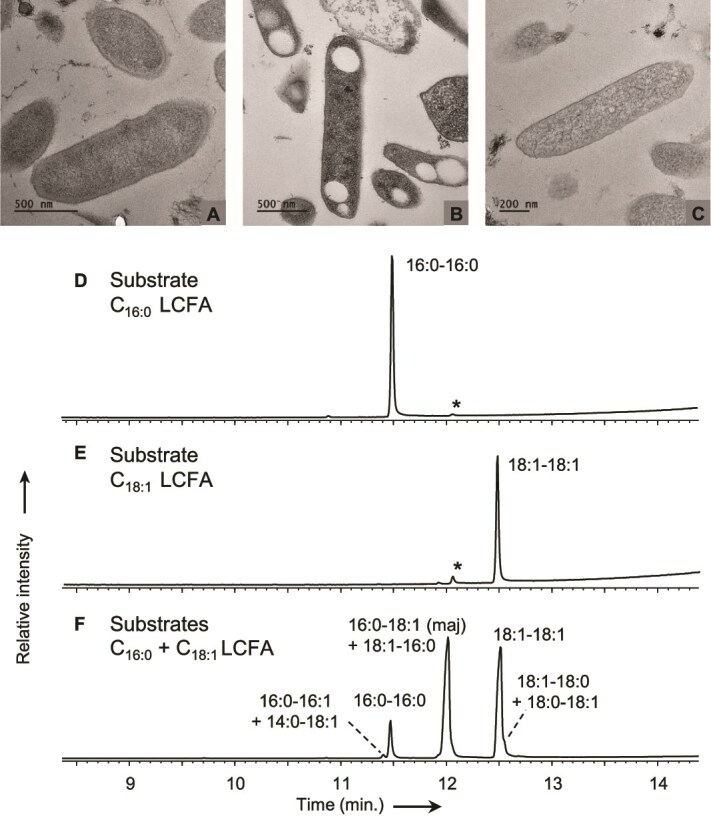
Evidence of WE production by *N. pharaonis*. (A–C) Transmission electron microscopy of *N. pharaonis* cells at the beginning (A) and after 10 days of incubation in nitrogen-limited medium supplemented with oleic acid (C_18:1_ LCFA) (B) or soluble substrates (acetate and pyruvate) (C). (D–F) GC-MS chromatograms of the WE fraction isolated from *N. pharaonis* cells grown on C_16:0_ (D), C_18:1_ (E) and a mixture of C_16:0_ and C_18:1_ (F) LCFA. WE are annotated as *x*:*x*′-*y*:*y*′ with *x* carbon atoms, *x*′ double bond in the alkyl chain, *y* carbon atoms, and *y*′ double bond in the acyl chain. * = contaminant.

The ability of *Halobacteriales* to synthesize WE and/or TAG *de novo* was investigated in one of the widespread extremely halophilic archaea possessing the WS/DGAT gene, *N. pharaonis.* In agreement with previous reports [43, 44], we observed that *N. pharaonis* grows well on individual LCFA or LCFA mixtures, in addition to the soluble substrates (acetate and pyruvate) conventionally used for growth. Transmission electron microscopy of thin sections of *N. pharaonis* after 10 days of incubation in N-limited medium supplemented with C_18:1_ LCFA revealed the presence of intracellular electron-transparent inclusion bodies, suggesting the accumulation of storage lipids (Fig. 3B). Similar inclusion bodies were rare at the beginning of incubation of *N. pharaonis* (Fig. 3A) in N-limited medium and absent during incubation with soluble substrates (pyruvate and acetate; Fig. 3C). The well-defined separation between each inclusion body formed by *N. pharaonis* (Fig. 3B) suggests that these inclusions possess a membrane-like structure that stabilizes the hydrophobic content of the body in the aqueous environment of the cytoplasm, as observed in bacteria. A generalized model proposed for the formation of neutral lipid bodies in bacteria suggests their initial formation at the plasma membrane and their progressive release in the form of mature lipid bodies localized in the cytoplasm [4, 7]. A similar mechanism could be envisaged in archaea, which would raise questions about the nature (isoprenoid vs. non-isoprenoid) of the membrane-like structure.

GC–MS analysis of neutral lipid extracts from *N. pharaonis* grown on LCFA demonstrated the production of WE (Fig. 3D–F). Growth on a single LCFA (C_16:0_ or C_18:1_) yielded a single WE with acyl and alkyl chains similar to those of the substrate (Fig. 3D and E), whereas growth on a mixture of C_16:0_ and C_18:1_ LCFA yielded WE composed of a saturated or monounsaturated C_14_ to C_18_ alcohol moiety esterified to a saturated or monounsaturated C_16_ to C_18_ acyl counterpart (Fig. 3F). In agreement with microscopic observations, WE were not detected during growth of *N. pharaonis* on soluble substrates such as acetate or pyruvate. The absence of WE synthesis during growth on soluble substrates, even under N-limiting conditions, supports the inability of *N. pharaonis* to synthesize LCFA *de novo*, as is generally considered for archaea [4]. The presence of WS/DGAT and the ability to synthesize WE supports the hypothesis that the WS/DGAT of *N. pharaonis* is responsible for WE biosynthesis, similarly to the WS/DGAT characterized in bacteria and eukaryotes [6, 8, 10]. However, whatever the culture conditions and growth phase, we never detected TAG in our GC–MS analyses, consistent with the absence of enzymes of the TAG biosynthesis pathway in *N. pharaonis* (Supplementary Fig. S4). This suggests that *N. pharaonis* WS/DGAT is not bifunctional (unlike the WS/DGAT of *A. baylyi*) and cannot synthesize TAG. This could also be the case for other *Halobacteriales*, which also lack several enzymes of the TAG biosynthesis pathway. The closely related species *N. moolapensis*, whose genome lacks *WS/DGAT* (Fig. 2A and B), showed no production of WE or TAG (in GC–MS analysis) or intracellular inclusion bodies (in transmission electron microscopy), whatever the growth conditions. This supports a link between the presence of WS/DGAT and the ability to synthesize WE.

We monitored the kinetics of WE production in *N. pharaonis* grown on C_18:1_ LCFA in individual replicate cultures, in parallel with cell numbering approximated by the number of 16S rRNA gene copies (Supplementary Fig. S5). Whereas we observed slight differences in WE quantity and cell number between replicates during the first few days of incubation, heterogeneity increased for both parameters with incubation time. The experimental setup was designed to optimize WE recovery but did not allow the determination of WE cell content, as cell numbers were not monitored in the same cultures as WE. Nevertheless, the average amount of C_18:1_-C_18:1_ WE produced increased over time, reaching levels two to six times higher after 10 days in N-limited conditions compared to non-limiting conditions (Supplementary Fig. S5A). The average number of cells also appeared to increase with incubation time, although less linearly than the amount of WE (Supplementary Fig. S5B). The accumulation of lipid inclusions in prokaryotes frequently occurs after the cessation of growth and division [1], as lipid accumulation is an energy-expensive process that competes with cell growth [6]. Thus, the continuous production of WE in *N. pharaonis* contrasts with the pattern of late-phase lipid accumulation reported in bacteria. The archaeal *WS/DGAT* gene is often included in the cluster of *atp* genes encoding ATP synthase or alongside *atpD* when separated from other *atp* genes, as is the case in *N. pharaonis* (Fig. 2C). This co-location suggests that *WS/DGAT* and ATP synthase genes are co-regulated and may respond to similar changes in cellular activity. This could explain why *WS/DGAT* expression levels remained consistent whether *N. pharaonis* was producing WE or not (i.e. in the presence of LCFA or soluble substrates, respectively; Supplementary Fig. S6). The regulation of WE synthesis in *Halovenus* species may differ because *WS/DGAT* is separated from *atp* genes in their genomes (Fig. 2C). The continuous production of WE observed in *N. pharaonis* may be due to the constitutive expression of *WS/DGAT*. This, combined with the inability of *N. pharaonis* to synthesize LCFA *de novo*, suggests that the WE biosynthesis pathway in *N. pharaonis* is regulated differently than in bacteria.

These results also strongly suggest that the archaeal WS/DGAT is functional despite its unusual NHXXXDG motif. They also suggest that in the absence of FarA, AcrB, and (most likely) Acr1, the WE synthesis pathway involves an as yet uncharacterized FcrA.

### Wax ester biosynthesis in archaea involves a 3-hydroxy-3-methylglutaryl-CoA reductase homolog as fatty acyl-CoA reductase, also present in numerous bacteria

**Figure 4:**
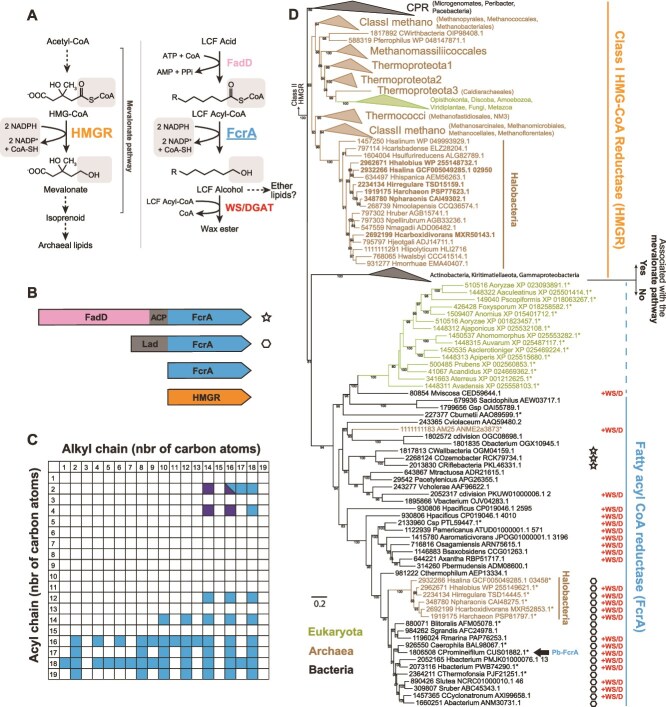
Identification of a FcrA evolutionarily related to HMGR. (A) Reactions catalyzed by the two enzymes in the context of their respective pathways: The mevalonate pathway (left) or the WE synthesis pathway (right). Dotted arrows indicate steps encoded by enzymes that are not shown. (B) Variability of protein domains associated with the FcrA core domain. (C) Lengths of alkyl and acyl carbon chains of WE formed by *E. coli* expressing Pb-fcrA and Ab-WS/DGAT and growing on glycerol, C_16:0_ or C_18:1_ LCFA; dark boxes indicate WE formed by *E. coli* grown on C_16:0_ LCFA and expressing Pb-fcrA without Ab-WS/DGAT. Corresponding GC–MS profiles are presented in Supplementary Fig. S8. (D) Maximum likelihood tree of Class-I HMGR and FcrA. Stars and hexagons next to FcrA sequences indicate the additional domains shown in (C). The arrow next to “Ca. Promineifilum” indicates the FcrA sequence (Pb-FcrA) expressed in *E. coli*. Sequence names marked with an asterisk in the FcrA/putative FcrA clade correspond to genomes that also possess Class-I HMGR and the mevalonate pathway. The other genomes in this clade do not possess the mevalonate pathway. *Halobacteriales* species corresponding to sequences highlighted in bold in the Class-I HMGR also possess FcrA. “+WS/D” indicates the presence of WS/DGAT in the corresponding taxon. The tree is based on a trimmed alignment of 302 amino acid positions and is rooted using Class-II HMGR as outgroup. Values on the tree indicate ultrafast bootstrap support >80%.

To identify the alternative FcrA involved in WE synthesis, we searched for protein families with a phyletic distribution pattern similar to that of WS/DGAT in *Halobacteriales* (Supplementary Table 3). The protein family most specifically co-occurring with WS/DGAT was annotated as a 3-hydroxy-3-methylglutaryl-CoA reductase (HMGR) Class-I. HMGR (Class-I and Class-II) catalyze the reduction of HMG-CoA to mevalonate in the mevalonate pathway [45], as part of the isoprenoid biosynthetic pathway (Fig. 4A). As isoprenoid units are the building blocks of archaeal lipids, HMGR is present in almost all archaeal genomes [46, 47]. The majority of *Halobacteriales* representatives with *WS/DGAT* in our dataset contain two copies of the *HMGR* gene. The presence of a secondary copy of this gene in *N. pharaonis* has previously been reported, and its function questioned [46]. In two *Halovenus* genomes, the gene encoding the secondary HMGR is located next to *WS/DGAT*, whereas in *Halovenus carboxidivorans* WSH3, a *fadD* gene is also located next to these two genes (Fig. 2C), thus constituting a complete pathway for WE synthesis from LCFA (Fig. 4A). This secondary *Halobacteriales* HMGR also possesses an additional N-terminal domain that is absent from the characterized HMGR (Fig. 4B), suggesting a different function. Furthermore, the four-electron reduction and removal of CoA catalyzed by HMGR corresponds to the reaction expected by the FcrA (Fig. 4A). The HMGR phylogeny built from archaeal, bacterial, and eukaryotic genomes, indicates that several HMGR-like bacterial proteins with the same N-terminal extension are closely related to the secondary HMGR of *Halobacteriales* (Fig. 4D). Most of these bacteria also possess a *WS/DGAT* for WE synthesis, and one of them (a *Holophagaceae* bacterium, contig PMJK01000076.1) has the *HMGR*-like gene next to *WS/DGAT*. These HMGR homologs with an N-terminal extension are part of a large and well-supported monophyletic group, distinct from the group containing the characterized HMGR of archaea and eukaryotes (Fig. 4D). Bacteria that possess the HMGR-like homolog and not the canonical homolog also lack other enzymes of the mevalonate pathway, arguing for a different role of this enzyme in archaea and bacteria. Taken together, these elements suggest that the secondary HMGR homologs in *Halobacteriales* and their closest bacterial counterparts represent an as yet uncharacterized type of fatty acyl-CoA reductase (that we named FcrA), involved in the four-electron reduction of fatty acyl-CoA to fatty alcohol.

One of these potential FcrA was expressed in *E. coli*. To avoid the difficulties associated with the expression of genes from extremely halophilic archaea in *E. coli* [48], we chose to express a gene from “Ca. Promineifilum breve” [49], a bacterium that grows under similar physicochemical conditions as *E. coli*. This potential FcrA, hereafter referred to as Pb-FcrA, belongs to the sister group of the potential FcrA in *Halobacteriales* (Fig. 4D) and share the same N-terminal extension than its archaeal relatives (Fig. 4B). Pb-FcrA was expressed with and without the *WS/DGAT* gene from *A. baylyi* (referred to as *Ab-WS/DGAT*). In the presence of palmitic acid (C_16:0_ LCFA), *E. coli* expressing *Pb-fcrA* without *Ab-WS/DGAT* accumulated C_16:0_ alcohol (Supplementary Fig. S7A) and produced a few WE composed of C_14:0_ or C_16:0_ alkyl chains esterified to acetate or butyrate (Fig. 4C and Supplementary Fig. S8D). Alcohol accumulation and limited WE production were not observed in *E. coli* transformed with an empty vector (Supplementary Figs S7B and S8E). Conversely, expression of *Pb-fcrA* in the presence of *Ab-WS/DGAT* with glycerol, C_18:1_ LCFA, or C_16:0_ LCFA as growth substrate yielded a series of WE with acyl and alkyl chains ranging from C_2_ to C_19_ and from C_1_ to C_18_, respectively, depending on the substrate (Fig. 4C and Supplementary Fig. S8A–C). Overall, these results show that *E. coli* cannot reduce fatty acids to fatty alcohols in the absence of Pb-FcrA and confirm that FcrA is a fatty acyl-CoA reductase involved in WE synthesis. The production of WE, albeit minimal, when *Pb-fcrA* is expressed in the absence of *Ab-WS/DGAT* is surprising. However, the marked contrast with WE production in the presence of *Ab-WS/DGAT* suggests that WE production without *Ab-WS/DGAT* is due to an unknown enzyme in *E. coli*, potentially carrying a non-specific reaction due to the accumulation of fatty alcohol produced by *Pb-fcrA*.

Enzymatic assays with purified Pb-FcrA revealed that it uses NADPH rather than NADH as electron donor, similarly to Class-I HMGR ([50]; Fig. 4A) and the fatty acyl-CoA reductases FarA and AcrB ([16, 17]; Fig. 1B). FcrA does not reduce a C_16:1_ aldehyde, unlike FarA and AcrB, and does not reduce HMG-CoA, unlike HMGR. Moreover, whereas HMGR is strongly inhibited by statins (*K*_i_ of the order of nM; e.g. [50, 51]), no inhibition of FcrA was observed with 10 μM simvastatin (free acid form). NADPH oxidation was not observed in the presence of LCFA-CoA with acyl chains shorter than 14 carbons, which is consistent with results from the GC–MS analysis of the neutral lipids of *N. pharaonis* grown on C_16:0_ and C_18:1_ LCFA (Fig. 3). This contrasts, however, with the heterologous expression of *Ab-WS/DGAT* and *Pb-fcrA* in *E. coli*, which produced WE with an alkyl chain shorter than 14 carbons (Fig. 4C and Supplementary Fig. S8).

**Figure 5:**
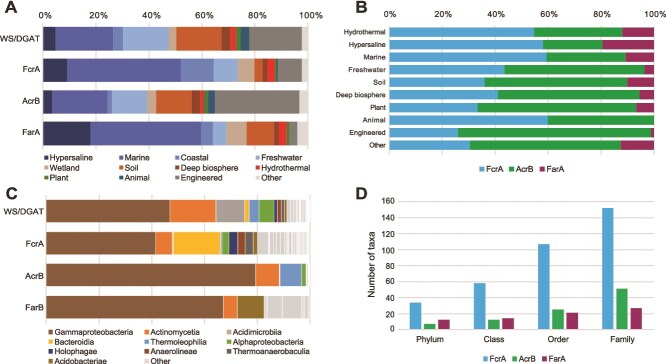
Environmental and taxonomic distribution of enzymes involved in WE synthesis. (A) Environmental distribution of WS/DGAT and the three fatty acyl-CoA reductases (FcrA, AcrB, and FarA). (B) Relative abundance of FcrA, AcrB, and FarA in different types of environments. (C) Taxonomic distribution of WS/DGAT, FcrA, AcrB and FarA. (D) Diversity of taxa possessing these enzymes. The analysis is based on the genomic catalog of Earth’s microbiomes [29].

We searched for the three fatty acyl-CoA reductases FarA, AcrB, and FcrA in our taxonomically balanced bacterial database (1093 bacteria covering all major phyla, Bac-1093; [28]). Among bacteria possessing WS/DGAT, 1% have FarA, 31% have AcrB, and 26% have FcrA, suggesting an important role for this latter enzyme in WE synthesis in bacteria. The analysis of the ca. 50 000 bacterial genomes from the Earth’s microbiomes catalog [29] further shows that FcrA is present in bacteria living in a wide range of environments (Fig. 5A and B). In particular, FcrA is more common than AcrB and FarA in (hyper)saline, hydrothermal, and animal microbiota, whereas AcrB is more widespread in other types of environments such as soil and freshwater (Fig. 5B). Like WS/DGAT, the three fatty acyl-CoA reductases are mainly found in *Gammaproteobacteria* and *Actinomycetia* (Fig. 5C). However, FcrA has a broader taxonomic distribution than the other two enzymes (Fig. 5C and D).

### Structural comparison between modeled fatty acyl-CoA reductase and human 3-hydroxy-3-methylglutaryl-CoA reductase

**Figure 6:**
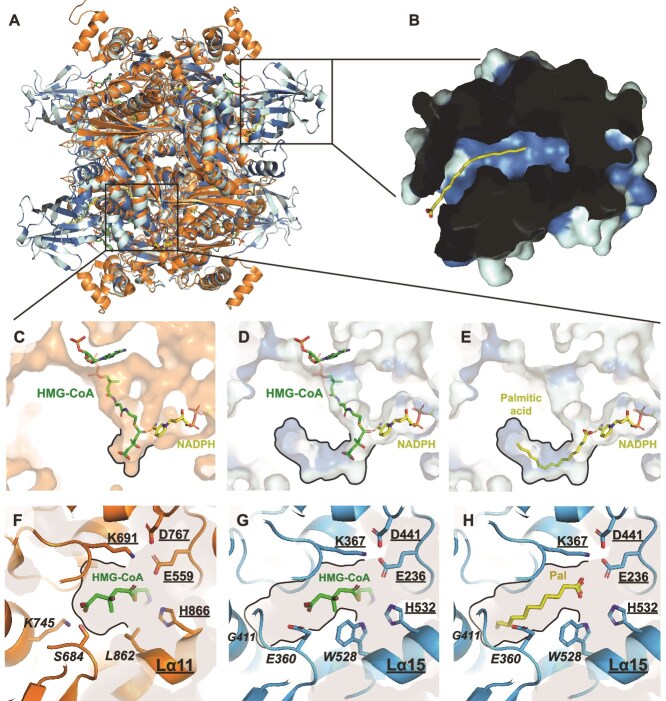
Comparison of the crystal structure of human HMGR (hHMGR, 1DQ9) with the FcrA AlphaFold 3 (AF3) structure model of *N. pharaonis*. (A) Superposition of HMGR and FcrA homotetramers. (B) Additional N-terminal domain of *N. pharaonis* FcrA (Lad) with palmitic acid (yellow) positioned by AF3. (C–E) comparison of the shape of the substrate-binding pocket of HMGR (C) and FcrA (D and E). (F–H) Catalytic and selected HMG-binding pocket residues of HMGR (F) and corresponding residues in FcrA (G and H). The catalytic residues and C-terminal alpha helix conserved between the two enzymes are underlined, whereas different residues in the HMG/alkyl-binding pocket are italicized. The shape of the HMG/alkyl-binding pocket is surrounded by a dark outline (C–H). NADPH and palmitic acid were positioned by AF3 in *N. pharaonis* FcrA, whereas HMG-CoA was co-crystallized with HMGR 1DQ9. For comparison, NADPH was represented in HMGR (C) and HMG-CoA in FcrA (D and G). Palmitic acid (C_16:0_ LCFA) was used as an indicator of the position of the palmitoyl-CoA carbon chain. Hydrophobic residues of FcrA are colored dark blue in panels (A, B, D, and E).

To investigate the structural similarities and differences between FcrA and HMGR, we modeled the structure of FcrA from *N. pharaonis* and *P. breve* using AlphaFold 3 and compared it to the crystal structure of human HMGR co-crystallized with HMG-CoA (hHMGR; 1DQ9; [52]). The predicted folding of the FcrA homotetramer is very similar to HMGR (Fig. 6A). The as yet undescribed N-terminal domain specific to certain FcrA (Figs 4B and 6A and B) consists of 10 β-sheets and is connected to the C-terminal part of the enzyme by an unstructured linker (Supplementary Figs S9 and S10). These β-sheets form a hydrophobic pocket that could accommodate a linear alkyl chain of more than 16 carbons (Fig. 6B). The role of this long-chain alkyl carrier domain (Lad) could be to transport (i) LCFA to FadD, (ii) fatty alcohols produced by FcrA to WS/DGAT, or (iii) fatty acyl-CoA to the catalytic site of FcrA. The acyl-carrier protein (ACP) domain fused to several bacterial FcrA (Fig. 4B and D) may play a role similar to Lad. Another major difference between HMGR and FcrA is the shape of the buried HMG/alkyl-chain substrate-binding pocket, which is inferred to be elongated in FcrA, allowing the accommodation of the long carbon chain of fatty-acyl CoA (Fig. 6C–E). In this analysis, palmitic acid (C_16:0_ LCFA) was used as an indicator of the position of the palmitoyl-CoA carbon chain. Structural modeling of the 59 potential FcrA enzymes used to construct the HMGR/FcrA tree (Fig. 4D) shows that a C_14_ carbon chain can fit 47 of them, mainly in bacteria and archaea (Supplementary Fig. S11), whereas 12 may accommodate smaller molecules, mainly in eukaryotes. As a control, the structure corresponding to 46 HMGR-affiliated sequences has also been modeled, revealing a small pocket that fits HMG.

FcrA exhibits a high degree of amino acid conservation around the catalytic site and within the hydrophobic pocket of the Lad domain (Supplementary Fig. S12). In particular, the catalytic residues involved in the reduction of HMG-CoA to mevalonate (Fig. 6F, Glu559, Lys691, Asp767, and His866 in human [52] and archaeal [50] HMGR) are highly conserved in all FcrA homologs, corresponding to Glu236, Lys367, Asp441, and H532 in *N. pharaonis* FcrA (Fig. 6G and H and Supplementary Fig. S9). This indicates that the reduction of fatty acyl-CoA by FcrA probably proceeds by a mechanism similar to the reduction of HMG-CoA by Class-I HMGR. In HMGR, the C-terminal helix Lα11 flips to close the catalytic pocket when the substrate is bound [50, 52]. This conformational change locks HMG-CoA and brings the conserved catalytic His866, located on the helix Lα11, close to the thiol group of HMG-CoA. The Lα11 C-terminal helix of hHMGR is conserved in FcrA (Lα15; Fig. 6G and H and Supplementary Fig. S9). In contrast, three highly conserved residues of the HMG-CoA-binding pocket (Ser684, Lys795, and Leu862 in hHMGR) are different in FcrA and may participate to substrate selection. Leu862, situated on the N-terminal side of the helix Lα11 in hHMGR (Fig. 6F), is replaced by a bulky amino acid in all FcrA (Trp528 on the Lα15 of *N. pharaonis*) (Fig. 6G and H and Supplementary Fig. S9). The function of Trp528 could be to stabilize the unbranched carbon chain of the acyl-CoA substrate and counter-select the branched chain of HMG by steric hindrance. The negatively charged Glu360 in *N. pharaonis* replaces Ser684 of HMGR and may also have a dual function in substrate selection. It may repel the negatively charged carboxylate group of HMG-CoA and stabilize the carbon chain of the acyl-CoA. Finally, the universally conserved Lys795 of HMGR is replaced by a variety of generally small amino acids in FcrA (Gly411 in *N. pharaonis*). This replacement removes a strong salt bridge interaction with HMG and likely participates to extend the substrate-binding pocket for long alkyl chains. These differences may also contribute to the lack of FcrA inhibition by simvastatins.

### Evolution of 3-hydroxy-3-methylglutaryl-CoA reductase Class-I and fatty acyl-CoA reductase

Our phylogeny of HMGR/FcrA (Fig. 4D) supports the previously proposed presence of the Class-I HMGR in the last common ancestor of Archaea [46, 47, 53]. In contrast to previous studies, eukaryotic sequences branch within Archaea, indicating that they originate from this domain. The closest sequences to the eukaryotic sequences are not those of *Asgardarchaeota* (which have a Class-II HMGR), but those of *Thermoproteota*, the closest lineage to *Asgardarchaeota*. All previously characterized Class-I HMGR belong to archaea and eukaryotes and are involved in mevalonate synthesis [50]. The evolutionary origin of bacterial Class-I HMGR is unclear [46, 47, 53], and none of these sequences have been characterized to date. It was previously suggested that some bacterial Class-I HMGR may have other functions than HMG-CoA reduction, as they are present in genomes that lack other genes of the mevalonate pathway [53]. Our findings support these initial predictions, showing that they are instead involved in the reduction of fatty acyl-CoA to fatty alcohol. Among the bacterial sequences previously annotated as Class-I HMGR, a large proportion correspond to FcrA (57% of our taxonomically balanced Bac-1093 database; 77% in the Earth’s microbiomes catalog [29]). Given that the FcrA sequences form a well-supported monophyletic clade, they could have emerged from a single event of neofunctionalization of a bacterial Class-I HMGR and subsequently spread by horizontal gene transfer among bacteria and *Halobacteriales*. However, their initial function may not have been linked to WE synthesis. Indeed, unlike *acrB*, which is almost exclusively present in genomes with *WS/DGAT* (94% of the time), *fcrA* and *farA* are less co-occurring with *WS/DGAT* in bacteria (Supplementary Fig. S13), indicating that they are regularly involved in pathways other than WE synthesis. The FarA domain is sometimes part of a large multifunctional protein (ElbD, also containing a FadD, an ACP and a PlsC-like domain, [54]) that is involved in the synthesis of alkyl-based glycerol ether lipids (plasmalogens) in *Myxococcales*. Some FcrA may have a similar role to FarA, providing fatty alcohols for ether lipid biosynthesis (Fig. 4A). Indeed, several bacterial taxa possessing FcrA also have a gene coding for a PlsC-like domain homolog involved in ether lipids synthesis. In the case of *Coxiella burnetii* RSA 493, this gene is located alongside *fcrA*. Other genomes coding for FcrA encode an alkylglycerone phosphate synthase (AGPS) that replaces the fatty acyl-group of 1-acyl-DHAP with a fatty alcohol. In “Ca. Promineifilum breve,” an *AGPS* gene is found nine genes after *Pb-FcrA*, which could indicate a dual role for *Pb-FcrA* in WE synthesis and ether lipid synthesis in this bacterium.

### Wax ester biosynthesis in the particular context of archaea

The narrow distribution of the WE biosynthetic pathway in archaea is probably linked to the fact that cellular accumulation of WE requires large quantities of LCFA (Fig. 1B; [6]). Unlike bacteria and eukaryotes, archaea do not require LCFA to synthesize their membrane lipids, which are synthesized from isoprenoid precursors. Although long debated, the ability of archaea to synthesize (LC)FA has recently been demonstrated in *Sulfolobus acidocaldarius* (*Thermoprotei*) and *Haloferax volcanii* using ^13^C-labeled substrates, and a new pathway was identified in *Thermoprotei* and *Asgardarchaeota* [55]. However, the amounts of FA produced by these archaea remain low [55], which may not be compatible with WE accumulation. In agreement, WE were not detected in *N. pharaonis* grown on acetate and pyruvate (Fig. 3C). In contrast, the presence of the WE biosynthetic pathway in members of the *Halobacteriales* may be linked to their potential, albeit still largely unexplored, ability to use LCFA as a source of carbon and energy. Indeed, although growth on LCFA has so far only been reported in *Natronomonas* species among the *Halobacteriales* ([56] and this study), many of these halophilic archaea have the genetic potential (*fadD* and β-oxidation pathway) to utilize these compounds (Fig. 2B). In addition, several members of the *Halobacteriales* can grow on long-chain alkanes [57, 58], implying that LCFA are used as metabolic intermediates in alkane degradation. The use of LCFA by some *Halobacteriales* may have facilitated the acquisition of the WE biosynthetic pathway by a member of this archaeal order. In particular, members of the family *Haloarculaceae*, where the WE biosynthetic pathway has been acquired, have a higher number of genes encoding FadD than other members of the *Halobacteriales* (Fig. 2B), which may reflect a more common utilization of LCFA. Outside the *Halobacteriales*, the ability to degrade LCFA via the β-oxidation pathway has been reported for *Archaeoglobus fulgidus* [59–61] and *S. acidocaldarius* [55] and proposed in several other uncultured lineages of anaerobic archaea [27, 62], but we did not identify WS/DGAT in these taxa.

### Potential physiological, ecological, and biotechnological implications

The specific functions of WE in *N. pharaonis* and other halophilic archaea have yet to be investigated, but some advantages of this metabolic capacity can be envisaged.

WE accumulation in the form of intracellular lipid bodies in prokaryotes generally serves to store energy and carbon, but other functions have been proposed for these neutral lipids [6, 7]. The ability to accumulate storage lipids requires maintaining a balance between precursors and reducing equivalents, and the accumulation of either type of precursor can become toxic to cells. Lipid bodies may thus act as a repository for toxic or useless fatty acids/alcohols during growth on specific carbon sources [11]. These aspects may be particularly relevant in the case of archaea that use LCFA as growth substrates, whose intracellular accumulation and/or their by-products could inhibit the proper functioning of the cell.

In addition, based on the presence of transporters for exogenous nitrogen sources, the genome of *N. pharaonis* suggests that this species is adapted to cope with reduced levels of ammonium ions [43]. This is in line with the extreme pH and high alkalinity conditions of the environments where *N. pharaonis* typically grows, which reduce the availability of ammonium ions. The increase in WE production observed under N-limited conditions (Supplementary Fig. S5) may support an adaptive capacity of the strain to cope with unbalanced carbon and nitrogen availability, as is classically observed in WE-producing bacteria [3, 6, 63].

Hypersaline environments are also characterized by strong osmotic gradients and evaporitic conditions. WE are water-insoluble, osmotically inert storage compounds. Nevertheless, the storage and consumption of evaporation-resistant lipids has been shown to contribute to cell survival under water stress conditions [64]. The formation of storage lipids may not only provide a source of carbon and energy but also constitute a basic metabolic supply of water through the esterification reaction, helping cells to survive under desiccating conditions. It has been suggested that this process is advantageous for halophilic microorganisms to adapt to and/or survive in low-energy, low-nutrient environments, such as evaporitic hypersaline environments and the deep biosphere [65, 66]. It has also been shown that WE accumulation contributes to the long-term survival (more than 360 days) and potential dormancy of some pathogenic bacteria such as *Mycobacterium tuberculosis* in the environment [67], inducing selective advantages in evolution, which could also be the case for halophilic archaea.

Furthermore, due to the preferential aerobic lifestyle of halophilic archaea, the production of intracellular lipid bodies may enable cells to control their buoyancy to cope with the strong oxygen gradients encountered in hypersaline environments (induced by the low solubility of oxygen in highly saline waters) and facilitate cell positioning in the more oxygenated parts of the brines. *Natronomonas* cells have been shown to be mobile, actively seeking optimal growth conditions with the help of retinal proteins [21]. However, unlike other *Halobacteriales* species that possess two sensory rhodopsin photosystems (SRI and SRII) mediating photo-attractant and photo-repellent responses, respectively [68], *N. pharaonis* possesses only the SRII photosystem [43, 69]. A link between the absence of the SRI photosystem and the potential ability to control cell buoyancy through WE production/consumption could be envisaged but remains hypothetical.

In addition to ecological and physiological advantages, the production and accumulation of WE by *N. pharaonis* reinforces the interest of halophilic archaea as microbial cell factories for the bioproduction of various marketable products, as previously demonstrated for bacterioruberin, bacteriorhodopsin, isoprenoids, and PHA [70]. Microbial lipids such as WE or fatty alcohols are potential resources for the sustainable production of value-added bioproducts and constitute an alternative and replacement pathway to petroleum-based chemicals [71, 72]. Their wide range of applications in fields such as pharmaceuticals, cosmetics, nutraceuticals, the environment, and biofuels support the development of low-cost, environmentally friendly production methods from renewable resources. Enzymes from extremophiles can be active and stable under harsh conditions, making them particularly interesting for biotechnological purposes. A variety of extracellular enzymes, including esterases and lipases, have been isolated from extremophilic archaea and used for a variety of medical and industrial purposes [73]. FcrA and WS/DGAT likely operating in *N. pharaonis* should exhibit high stability and optimal activity at high salt concentrations and pH values. This could give them stability in low-water environments, such as those encountered in biocatalytic reactions carried out in organic solvents [74].

In summary, our study shows that certain extremophilic archaea of the order *Halobacteriales* can synthesize neutral lipids *de novo* from non-isoprenoid LCFA, although archaea conventionally synthesize isoprenoid lipids. To produce the fatty alcohols required for WE synthesis, *Halobacteriales* reduce LCFA substrates to the corresponding alcohols using a fatty-acyl CoA reductase (FcrA) evolutionarily related to Class-I HMGR (classically involved in isoprenoid biosynthesis) and widely distributed in bacteria and the environment. Fatty alcohols and acids are then condensed into WE, probably by an archaeal WS/DGAT, which was most likely acquired by horizontal gene transfer from halophilic bacteria. FcrA and WS/DGAT are present in halophilic archaea isolated from various types of hypersaline environments, including saline lake water and sediments, soil, desert salt crust, or solar salterns, indicating that WE synthesis could be beneficial to *Halobacteriales* in a wide range of conditions. Most archaeal WS/DGAT homologs have an unusual NHXXXDG motif and may be specialized in WE synthesis (as suggested by the absence of genes of the TAG pathway). Characterization of this enzyme is necessary to validate its function and may provide broader information on the functioning of WS/DGAT enzymes and on what dictates their specificity for WE and/or TAG synthesis. More generally, by extending the possibility of WE biosynthesis to all domains of life, this study lays the foundation for further exploration of fatty acid-based lipid production in biological systems markedly different from eukaryotes and bacteria. It suggests specific physiological and ecological roles for WE in extremophilic archaea and provides an unprecedented basis for biotechnological applications involving (poly)extremophilic archaeal systems.

## Supplementary Material

ISME_Supplementary_information_Grossi_revised3_wraf035

## Data Availability

All data generated or analyzed during this study are included in this published article (and its supplementary information files).
