# Supplementary material for: Halophilic archaea produce wax esters and use an alternative fatty acyl-coenzyme A reductase for precursor synthesis
Source: ISME J. 2025 Feb 24;19(1):wraf035. doi: 10.1093/ismejo/wraf035 (PMC11948995; doi:10.1093/ismejo/wraf035)
Supplement: ISME_Supplementary_information_Grossi_revised3_wraf035 [file isme_supplementary_information_grossi_revised3_wraf035.pdf]

Supplementary Information for

Halophilic archaea produce wax esters and use an alternative fatty acyl-CoA reductase for precursor synthesis

Vincent Grossi*, Philippe Cuny, Cécile Militon, Jerzy Witwinowski, Balkis Eddhif, Léa Sylvi, Mireille Nowakowski, Artemis Kosta, Ingrid Antheaume, Johan Cornil, Sarah Dubrac, Julia Kende, Simonetta Gribaldo, Guillaume Borrel*

*Authors for correspondence: Vincent Grossi and Guillaume Borrel
Email adresses: vincent.grossi@mio.osupytheas.fr; guillaume.borrel@pasteur.fr

This file contains:
Additional information on the material and methods,
Figure S1 to S13
Supplementary Tables 1 to 4
References used in the supplementary methods and figures

Supplementary Methods

Natronomonas medium preparation and growth parameters

All chemicals were ordered from Sigma-Aldrich. DSM medium 371 (which we termed N-rich) was used to grow *N. pharaonis* and *N. moolapensis*. The medium was prepared using (gram per liter distilled water): KH_2PO_4 (1), KCl (1), NH_4Cl (1), $MgSO_4.7H_2O$ (0.24), $CaSO_4.2H_2O$ (0.17), SL-10 trace element solution (1ml), NaCl (200), Na_2-glutamate (1), yeast extract (5), casamino acids (5), Na_2CO_3 (5). The SL-10 trace element solution consisted of (g/l) HCl (2.5), FeCl2.$_4$H2O (1.5), $ZnCl_2$ (0.07), $MnCl_2.4H_2O$ (0.1), H_3BO_3 (0.006), $CoCl_2.6H_2O$ (0.19), $CuCl_2.2H_2O$ (0.002), $NiCl_2.6H_2O$ (0.024), $Na_2MoO_4.2H_2O$ (0.036). The pH was adjusted to 6.5 and the medium was autoclaved at 120 °C for 20 min. Na_2CO_3 was kept separated from the medium and added after cooling. The final pH was adjusted to 9.0-9.5.
The growth of the two *Natronomonas* strains was monitored on replicate cultures in N-rich medium, enabling generation rates to be calculated. The generation rate of *N. pharaonis* and *N. moolapensis* was around 110 and 40 minutes, respectively.

Cell numbering

The number of cells in each culture of *N. pharaonis* was estimated by measuring the copy number of the archaeal 16S rRNA gene from extracted DNA [1]. Q-PCR was performed using the GoTaq® qPCR Master Mix (Promega) according to manufacturer's protocol with the primers set 931F and 1100R (Supplementary Table 1). The real-time PCR cycles consisted of an initial denaturation at 98 °C for 3 min, followed by 35 cycles of denaturation at 98 °C for 10 s, hybridization at 62 °C for 10 s and elongation at 72 °C for 20 s. To verify the specificity of the system, a melting curve analysis was performed at the end of each PCR cycle. Standard curves were constructed with 3.10^8 to 30 copies of a pGEM-t Easy plasmid containing a Euryarchaeota insert. The resulting conditions led to a Q-PCR efficiency of 103% (R^2=0.997).

Electron Microscopy

Cell pellets were high-pressure frozen (Leica EM HPM100), freeze-substituted, and embedded in Epon resin (Medium Grade) according to [2]. Ultrafine sections (60-90 nm) were cut with an ultracryomicrotome (Leica EM UC7) and stained with uranyl

acetate and lead citrate [3]. The samples were analyzed using a Tecnai 200KV electron microscope (FEI), and digital acquisitions were made with a 4Kx4K CMOS camera (OneView Gatan).

Analysis of neutral lipids by GC and GC-MS

GC analyses of neutral lipid fractions were performed on a HP-6890 Series gas chromatograph equipped with a cool on-column injector and a flame ionization detector (GC-FID). GC-MS analyses were performed on a HP 6890 Series Plus gas chromatograph equipped with a cool on-column injector and coupled to an Agilent 5975C mass spectrometer (VL MSD). Compound separation was achieved using a high-temperature fused silica capillary column (15 m × 0.25 mm) coated with OPTIMA 5HT (0.10 µm film thickness) or an HP-5MS capillary column (30 m × 0.25 mm x 0.25 µm film thickness), using a constant flow (1 $ml.min^{-1}$) of helium as carrier gas. The GC oven temperature was increased from 70 °C (held 0.5 min) to 350 °C (held 20°C min-1) at 20 °C min^{-1}. Electron impact mass spectra were recorded at 70 eV in full scan mode in the m/z range 50-700. WE were identified based on their characteristic mass spectral fragmentations and by comparison with literature data [4], and quantified by GC using an external calibration with WE standards.

***Natronomonas pharaonis* DNA recovery**

DNA extraction was performed from each filter using a phenol-chloroform procedure. 5 ml extraction buffer (50 mM EDTA, 250 mM sodium acetate; pH 8.0), 5 ml phenol (pH 8.0), 0.5 ml SDS (20%, v/w) and a lysing matrix E (MP Biomedicals) were added to each filter in a tube and vortexed for 1 min. The samples were then manually shaken for 30 s and centrifuged for 10 min at 5,500 x g (4 °C). The aqueous phase from each tube was transferred to a new 15 ml tube. The pellet and organic phase were re-extracted with 300 µl of extraction buffer, vortexed for 1 min, shaken for 30 s, and centrifuged for 10 min (5,500 x g) at 4 °C. The combined supernatants were mixed with 1 V phenol (pH 8.0), vortexed and centrifuged for 10 min (2,500 x g) at 4 °C. The aqueous phases were mixed with 1 V phenol/chloroform (1:1), vortexed for 5 s, and centrifuged at 2,500 × g for 10 min (4 °C). The aqueous phase was then mixed with 1V chloroform, vortexed for 5 s, and centrifuged at 2,500 × g for 10 min (4 °C). The aqueous phase containing DNA was mixed with 2 V ethanol and 0.1 V ammonium acetate (5 M) and incubated at -20°C for 2 h. DNA was recovered by

centrifugation at 17,000 x g for 20 min (4 °C), washed with 70% ethanol, centrifuged at 17,000 × g for 20 min (4 °C) and resuspended in 50 µl of nuclease-free water.

RNA extraction and reverse-transcription

RNA extraction was performed on 10 ml of culture of *N. pharaonis* in N-limited media supplemented with oleic acid ($C_{18:1}$ LCFA) or soluble substrates (SS). The same procedure as for the DNA extraction was used with extraction buffer (pH 5) and phenol (pH 4.3). A desalting step was added at the end of the RNA extraction protocol using the MEGAclear RNA purification kit (Ambion), allowing efficient removal of DNA using the Turbo DNA-free kit (Ambion). Total denatured RNA (10 µl) was transcribed into cDNA using the SuperScriptIII kit (Invitrogen) according to the manufacturer's recommendations and 1µl of random primers.

Real-time PCR on ws/dgat genes

Expression of the *WS/DGAT* gene in each culture of *N. pharaonis* DSM2160 was estimated by qPCR using the same procedure as that used to estimate 16S copy number. To determine the number of *WS/DGAT* transcripts, 2 µl of cDNA and primers F_WS/DGAT_DSM2160 and R_WS/DGAT_DSM2160 were used. Standard curves were constructed with 5×10^9 to 5×10^2 copies of a pGEM-t Easy plasmid containing *atfA* from strain DSM 2160. The resulting conditions led to a qPCR efficiency of 81.4% (R^2=0.994).

***Escherichia coli* strains for gene expresssion**

E. coli strains used in this work are listed in Supplementary Table 3. These strains were genetically manipulated using standard laboratory procedures [5]. When needed, ampicillin (100 mg/l) or kanamycin (25-50 mg/l) was added to *E. coli* cultures.

DNA manipulations for gene expression

PCR reactions for cloning applications were carried out using Phusion HiFi Master Mix (Thermo Fisher Scientific) according to manufacturer’s protocol. PCR reactions for the control of constructions were carried out using the DreamTaq Green MasterMix (Thermo Fisher Scientific). Restriction enzymes were of the FastDigest family of products (Thermo Fisher Scientific). Digestion and PCR products were

isolated on agarose gels and purified with the GeneJET Gel Extraction kit (Thermo Fisher Scientific). Plasmid isolation was performed with NucleoSpin Plasmid kit (Macherey-Nagel). Sanger sequencing was performed by Eurofins. Sequence in silico manipulation was carried out using SnapGene (GSL Biotech, www.snapgene.com). Primers were designed with NEBuilder (New England Biolabs, https://nebuilder.neb.com/) or Primer3Plus [6]. Cloning was performed using NEBuilder HiFi DNA Assembly Master Mix (New England Biolabs) unless stated otherwise. *E. coli* cells were transformed according to [7]. For transformation of cloning products, the DH5α strain was used.

Protein expression and purification of Pb-FcrA

Pb-fcrA was amplified using JW397/JW398 primer pair and cloned into pT7-SUMO linearized by PCR using pT7-F/pT7-R primer pair, yielding pJW113 vector. Insert integrity was verified by Sanger sequencing.

Pb-fcrA was expressed from pJW113 in *E. coli* C43(DE3) (Merck, reference CMC0019) and purified using nickel-affinity chromatography followed by size exclusion chromatography. The cells were grown in one-liter 2YT broth with 50 µg/ml kanamycin at 37 °C and induced with 0.5 mM isopropyl β-D-1-thiogalactopyranoside (IPTG) at an Abs600 of 3. After further incubation at 30 °C for 4 h, the cells were harvested by centrifugation at 17000 × g -15 min. The cell pellet was resuspended in buffer A (50 mM phosphate (pH 7.5), 300 mM NaCl, 10 mM imidazole, 2 mM DTT, 10% glycerol), disrupted using a CELLD disruptor (Constant system) and clarified by centrifugation at 45 000 × g during one hour at 4 °C. The soluble fraction was then applied to a 1 ml nickel-chelating column (IMAC NICKEL Metal Affinity Resin, Macherey-Nagel). HMGR-like was eluted using a gradient of 25–300 mM imidazole in buffer A. Removal of SUMO tag was performed with SUMO protease in buffer A without imidazole 2 hours at 30 °C and the SUMO tag has been then eliminated with nickel affinity.

Concentrated flow-through fractions were then applied to a 16/60 Superdex 200 column (Cytiva) and developed with 50 mM Phosphate (pH 7.5), 300 mM NaCl, DTT 2 mM, glycerol 10%. At the end, DTT was added to the purified fractions at a final concentration of 10 mM before storage at -80 °C.

Simvastatin activation

To obtain free acid form of simvastatin, 8.4 mg of simvastatin (lactone form) was dissolved in 200 µl of pure ethanol, supplemented with 30 µl of 1 M sodium hydroxide, then incubated 2 h at 50°C. The pH was neutralized with 30 µl of 1 M hydrochloric acid, then 740 µl of water and 1 ml of DMSO were added.

Western blot

Bacterial cells were lysed using BugBuster protein extraction reagent (Merck), then mixed with an equal volume of 2x reducing sample buffer (prepared by mixing 5 ml of Novex LDS sample buffer 4x (Thermo Fisher Scientific), 4.5 ml of water and 0.5 ml of beta-mercaptoethanol). Gel electrophoresis was performed on a BOLT Bis-Tris Plus 4-12% gel (Thermo Fisher Scientific) in NuPAGE MES SDS buffer (Thermo Fisher Scientific) at 100 V. Proteins were transferred to a nitrocellulose membrane using the Power Blotter system (Thermo Fisher Scientific). The membrane was briefly washed with water, then incubated in 5% skimmed milk (Roth) dissolved in phosphate-buffered saline with 0.05% Tween-20 (PBST) for one hour. Two 5-min washes in PBST were performed, then the membrane was incubated in a mouse anti-6His-tag monoclonal gamma immunoglobulin (Thermo Fisher Scientific, reference MA-135) diluted to 200 µg/l in PBST containing 1% milk for one hour. A 5 min-wash was performed in 5% milk-PBST, two 5-min washes in PBST, then the membrane was incubated in a goat anti-mouse IgG polyclonal gamma immunoglobulin coupled to horse radish peroxidase (Thermo Fisher Scientific, reference 31430) diluted to 80 µg/l in 1% milk-PBST for one hour. Four 5 min-washes were performed in PBST, as well as a brief wash with water, and the western blot was revealed by the Amersham ECL kit (GE Healthcare).

SUPPLEMENTARY FIGURES

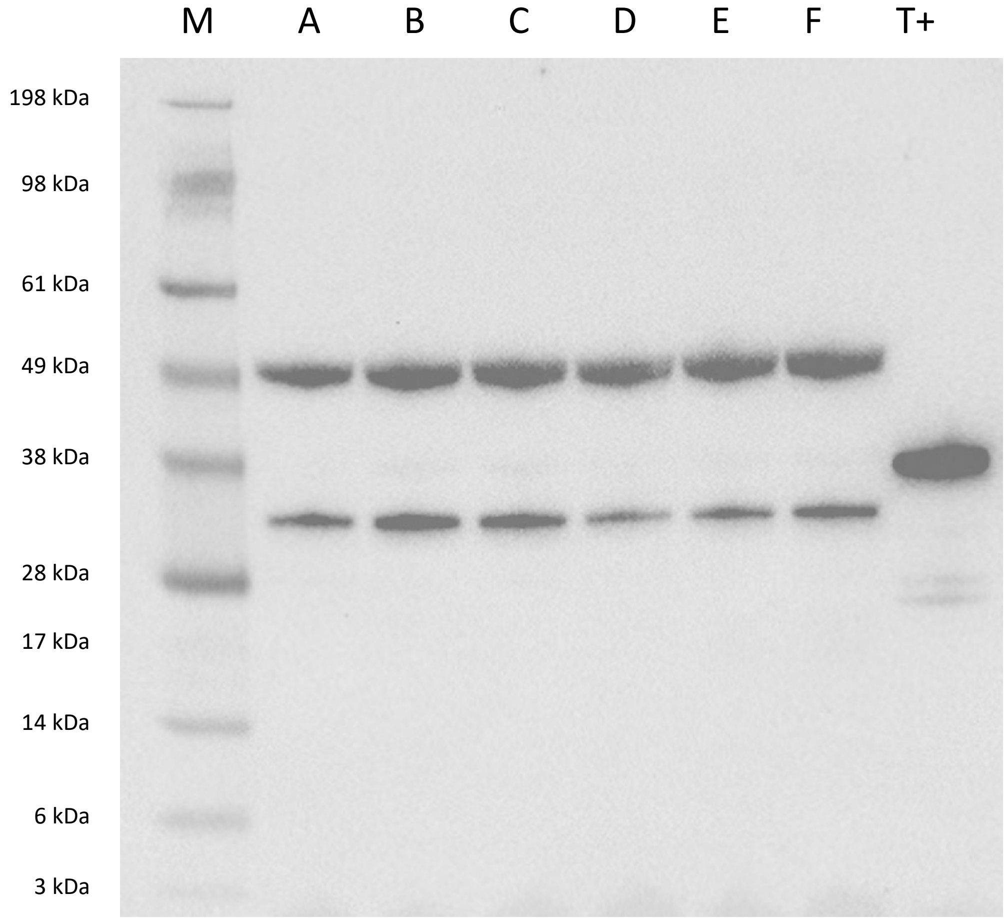

Figure S1: Western blot revealing the expression of His-tagged WS/DGAT from vector pJW105 (containing an artificial *Pb-fcrA – Ab-WS/DGAT* operon) with anti-6His tag antibody in *E. coli* strain BL21. M = Seeblue Plus2 Prestained Standard; A-C = 24h cultures, D-F = 48h cultures; A, D = cultures supplemented with glycerol; B, E = cultures supplemented with $C_{18:1}$ LCFA; C, F = cultures supplemented with $C_{16:0}$ LCFA; T+ = positive control (the same *E. coli* strain expressing a different His-tagged protein). The predicted molecular weigth of WS/DGAT-6His is 52.9 kDa.

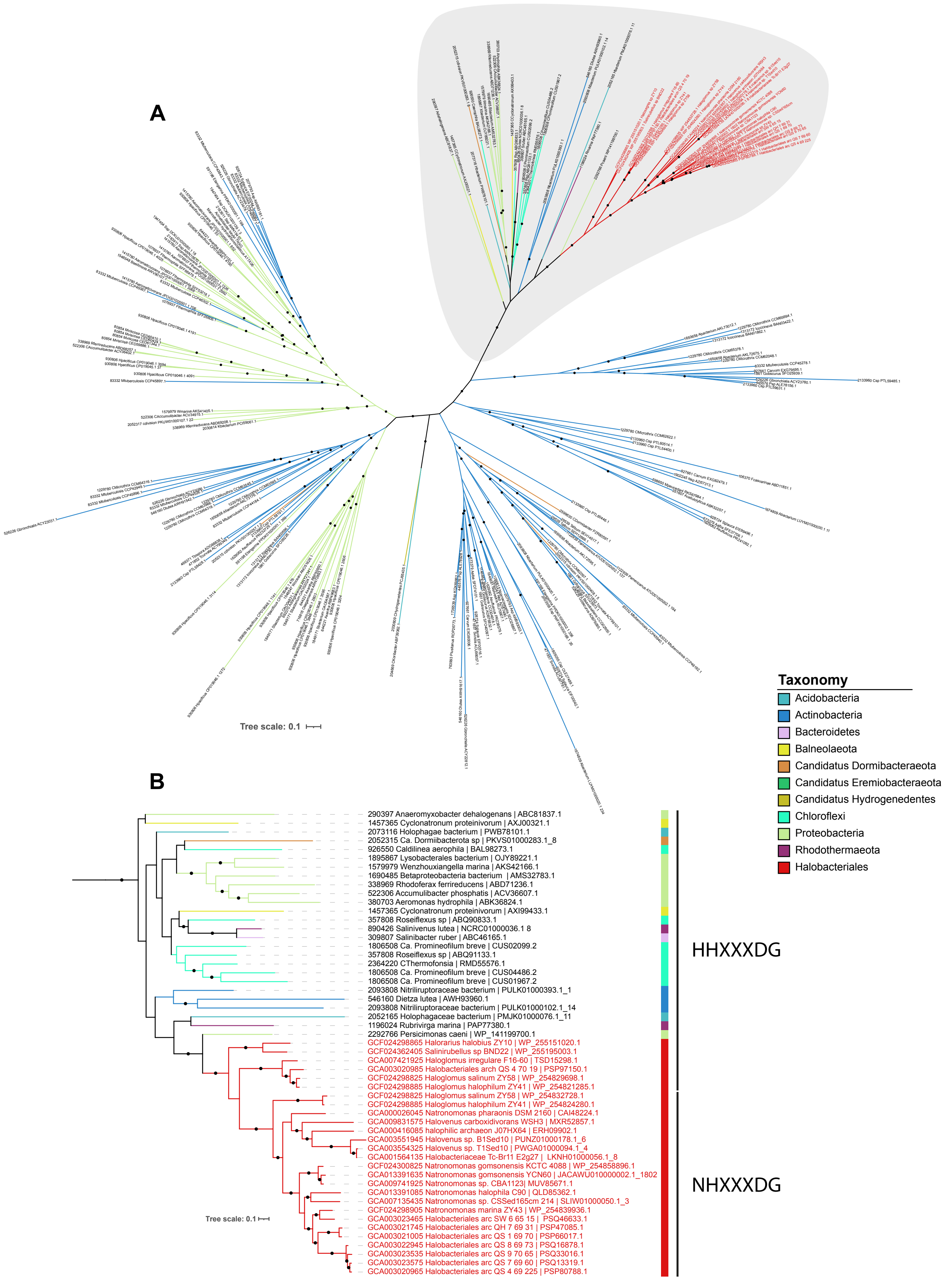

Figure S2: Phylogeny of WS/DGAT enzymes. (**A**) Maximum-likelihood tree (LG+F+R5) based on 205 WS/DGAT sequences, including 27 sequences of *Halobacteriales*. Node supports refer to ultrafast bootstrap values, only values above 0.9 are shown. (**B**) Subtree corresponding to sequences highlighted by a grey frame on panel A. HHXXXDG and NHXXXDG indicate the presence of these motifs in the corresponding sequences. *D. lutea* has a motif QHXXDG.

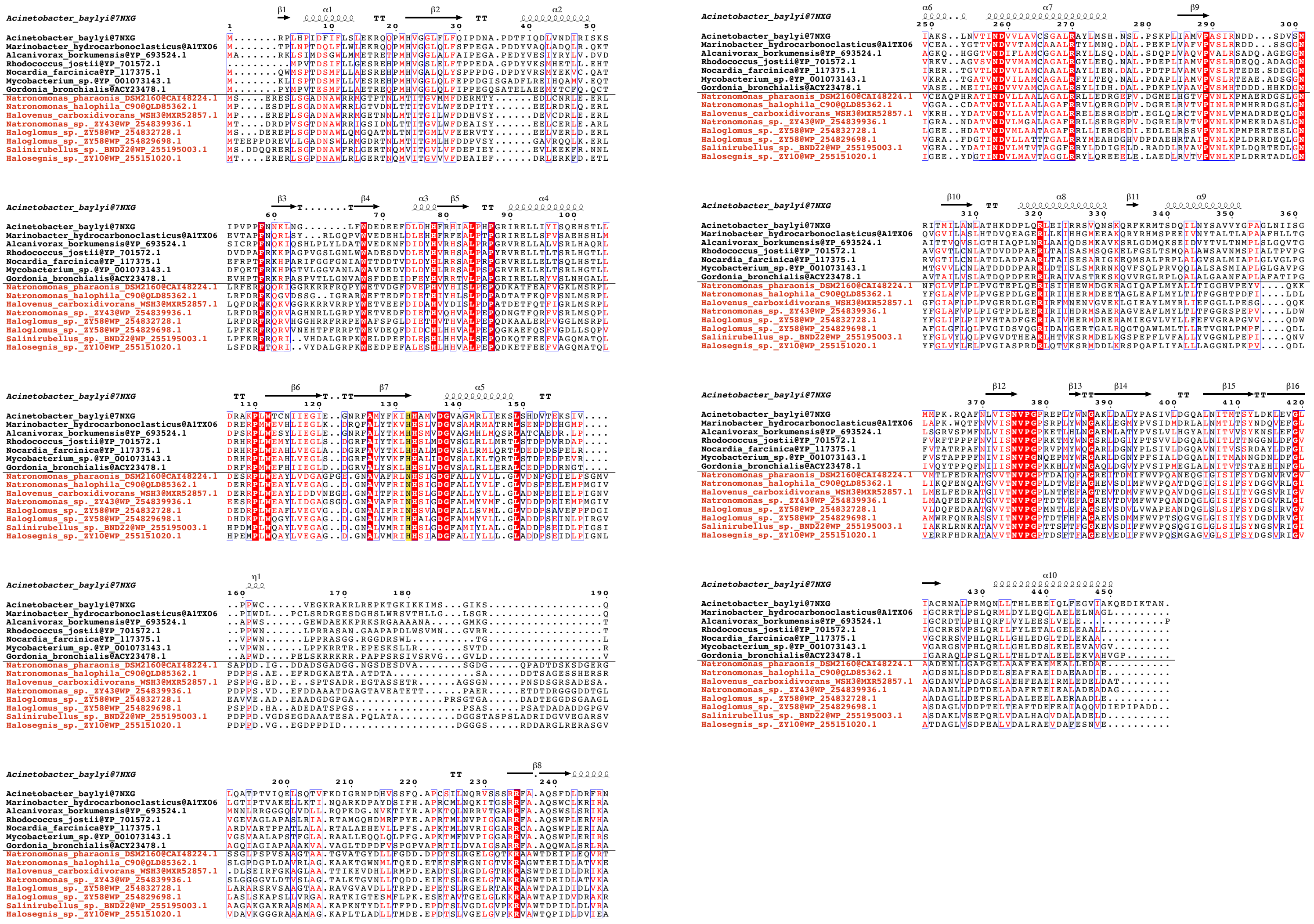

Figure S3. Sequence conservation between bacterial and archaeal (names in dark red) WS/DGAT, generated with the Espript server. His/Asn132 is highlighted in yellow.

A

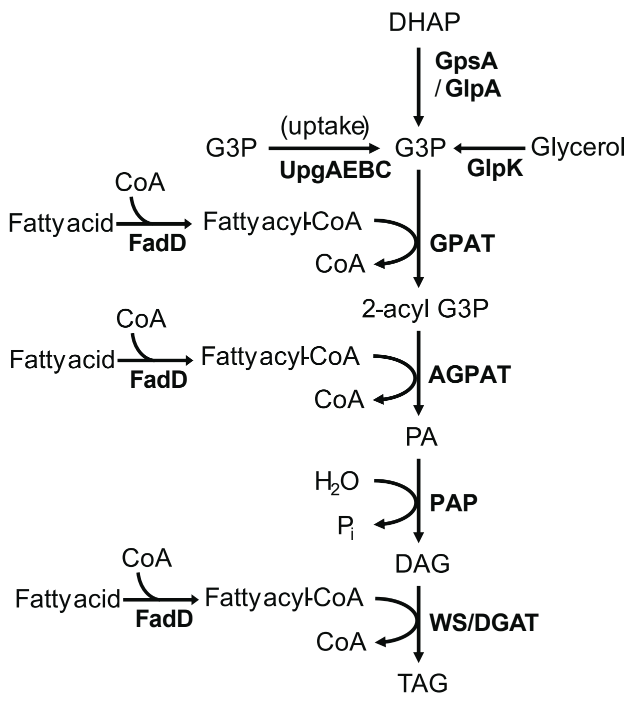

B

Assembly	Family	Species	WS/(DTAG)	GlpA/D	GlpK	UgpBAEC	GpsA	GPAT	AGPAT	PAP
GCA000026045	Haloarculaceae	Natronomonas pharaonis DSM 2160	+	-	-	-	-	-	-	-
GCA001564135	Haloarculaceae	Halobacteriaceae archaeon Tc-Br11_E2g27	+	-	-	-	-	-	-	-
GCA003021325	Haloarculaceae	Halobacteriales archaeon QH_10_70_21	+	-	-	+	-	-	-	-
GCA003021745	Haloarculaceae	Halobacteriales archaeon QH_7_69_31	+	+	+	+	-	-	-	-
GCA003021005	Haloarculaceae	Halobacteriales archaeon QS_1_69_70	+	-	-	+	-	-	-	-
GCA003020965	Haloarculaceae	Halobacteriales archaeon QS_4_69_225	+	+	-	+	-	-	-	-
GCA003020985	Haloarculaceae	Halobacteriales archaeon QS_4_70_19	+	+	+	-	-	-	-	-
GCA003023575	Haloarculaceae	Halobacteriales archaeon QS_7_69_60	+	-	-	-	-	-	-	-
GCA003022945	Haloarculaceae	Halobacteriales archaeon QS_8_69_73	+	+	+	+	-	-	-	-
GCA003023535	Haloarculaceae	Halobacteriales archaeon QS_9_70_65	+	+	-	-	-	-	-	-
GCF007421925	Haloarculaceae	Haloglomus irregulare F16-60	+	+	+	+	-	-	-	-
GCF000416085	Haloarculaceae	Halophilic archaeon J07HX64	+	+	+	-	-	-	-	-
GCF009831575	Haloarculaceae	Halovenus carboxidivorans WSH3	+	+	+	-	-	-	-	-
GCA003551945	Haloarculaceae	Halovenus sp. B1Sed10_163	+	+	-	-	-	-	-	-
GCA003554325	Haloarculaceae	Halovenus sp. T1Sed10_210R1	+	-	-	+	-	-	-	-
GCF013391635	Haloarculaceae	Natronomonas gomsonensis YCN60	+	+	+	-	-	-	-	-
GCA013391085	Haloarculaceae	Natronomonas halophila C90	+	+	+	-	-	-	-	-
GCF009741925	Haloarculaceae	Natronomonas sp. CBA1123	+	+	+	-	-	-	-	-
GCA007135435	Haloarculaceae	Natronomonas sp. CSSed165cm_214	+	-	-	+	-	-	-	-
GCF024298825	Haloarculaceae	Haloglomus salinum ZY58	+	+	+	-	-	-	-	-
GCF024298885	Haloarculaceae	Haloglomus halophilus ZY41	+	+	+	-	-	-	-	-
GCF024298865	Haloarculaceae	Halorarius halobius ZY10	+	+	+	+	-	-	-	-
GCF024362405	Haloarculaceae	Salinirubellus sp. BND22	+	+	+	+	-	-	-	-
GCF024298905	Haloarculaceae	Natronomonas marina ZY43	+	+	+	-	-	-	-	-
GCF024300825	Haloarculaceae	Natronomonas gomsonensis KCTC 4088	+	+	+	-	-	-	-	-
GCA003023465	Haladaptataceae	Halobacteriales archaeon SW_6_65_15	+	-	+	+	-	-	-	-
GCF005310945	Haloarculaceae	Haloarcula marismortui ATCC 43049	-	+	+	+	-	-	-	-
GCA003021175	Haloarculaceae	Halobacteriales archaeon QH_7_66_36	-	+	+	+	-	-	-	-
GCA009791395	Haloarculaceae	Halomarina oriensis JCM 16495	-	+	+	+	-	-	-	-
GCA000023965	Haloarculaceae	Halomicrobium mukohataei DSM 12286	-	+	+	+	-	-	-	-
GCA000023945	Haloarculaceae	Halorhabdus utahensis DSM 12940	-	+	+	-	-	-	-	-
GCF005049285	Haloarculaceae	Halorientalis salina NEN8	-	+	+	-	-	-	-	-
GCA016065055	Haloarculaceae	Halosimplex litoreum YGH94	-	+	+	+	-	-	-	-
GCF000591055	Haloarculaceae	Natronomonas moolapensis 8	-	+	+	-	-	-	-	-
GCF013391105	Haloarculaceae	Natronomonas salina YPL13	-	+	+	+	-	-	-	-
GCF009831455	Haloarculaceae	Salinirussus salinus YGH44	-	+	+	+	-	-	-	-
GCF020700235	Haladaptataceae	Haladaptatus pallidirubidus YIM 93656	-	+	+	-	-	-	-	-
GCF004087835	Haladaptataceae	Halorussus pelagicus RC-68	-	+	+	-	-	-	-	-
GCA000196895	Halalkalicoccaceae	Halalkalicoccus jeotgali B3	-	+	+	+	-	-	-	-
GCF004799665	Haloarculaceae	Halapricum salinum CBA1105	-	+	+	+	-	-	-	-
GCA000006805	Halobacteriaceae	Halobacterium salinarum NRC-1	-	+	+	+	-	-	-	-
GCF001886955	Halobacteriaceae	Halodesulfurarchaeum formicicum HSR6	-	+	-	-	-	-	-	-
GCF020614395	Halobacteriaceae	Salarchaeum japonicum JCM 16327	-	+	+	-	-	-	-	-
GCA003021725	Halococcaceae	Halobacteriales archaeon QH_8_64_26	-	+	+	+	-	-	-	-
GCA003021045	Halococcaceae	Halobacteriales archaeon QS_4_69_34	-	+	+	-	-	-	-	-
GCF009900715	Halococcaceae	Halococcus salsus ZJ1	-	+	+	+	-	-	-	-
GCA000495475	Haloferacaceae	Candidatus Halobonum tyrrellensis G22	-	+	+	-	-	-	-	-
GCF002844195	Haloferacaceae	Halegenticoccus soli SYSU A9-0	-	+	+	-	-	-	-	-
GCF902410085	Haloferacaceae	Haloferax sp.	-	+	+	-	-	-	-	-
GCF003605635	Haloferacaceae	Halonotius pteroides CECT 7525	-	+	+	+	-	-	-	-
GCF001462205	Haloferacaceae	Haloparvum sedimenti DYS4	-	+	+	+	-	-	-	-
GCF000009185	Haloferacaceae	Haloquadratum walsbyi DSM 16790	-	+	+	+	-	-	-	-
GCA000022205	Haloferacaceae	Halorubrum lacusprofundi ATCC 49239	-	+	+	+	-	-	-	-
GCA900103505	Natrialbaceae	Haloarchaeobius iranensis EB21	-	+	+	+	-	-	-	-
GCA000025325	Natrialbaceae	Haloterrigena turkmenica DSM 5511	-	+	+	+	-	-	-	-
GCA000328525	Natrialbaceae	Halovivax ruber XH-70	-	-	-	+	-	-	-	-
GCA000025625	Natrialbaceae	Natrialba magadii ATCC 43099	-	+	+	+	-	-	-	-
GCA017094485	Natronoarchaeaceae	Natranaeroarchaeum sulfidigenes AArc-S	-	+	+	+	-	-	-	-
GCA003021085	PXRE01	Halobacteriales archaeon QS_1_68_20	-	-	-	+	-	-	-	-
GCF005954745	QS-9-68-17	Halostella pelagica DL-M4	-	+	+	+	-	-	-	-
GCF000403645	Salinarchaeaceae	Salinarchaeum sp Harcht-Bsk1	-	+	+	+	-	-	-	-
GCA003023195	SW-7-71-33	Halobacteriales archaeon QS_8_69_26	-	-	-	+	-	-	-	-
GCA009889635	UBA12382	Marine Group IV archaeon HikBin1	-	-	-	-	-	-	-	-
GCA009889625	UBA12382	Marine Group IV archaeon HikBin5	-	+	-	-	-	-	-	-

Figure S4: Pathways and enzymes involved in TAG synthesis in bacteria (**A**) and presence/absence of these different enzymes in *Halobacteriales* (**B**). The FadD distribution is shown in Figure 2B.

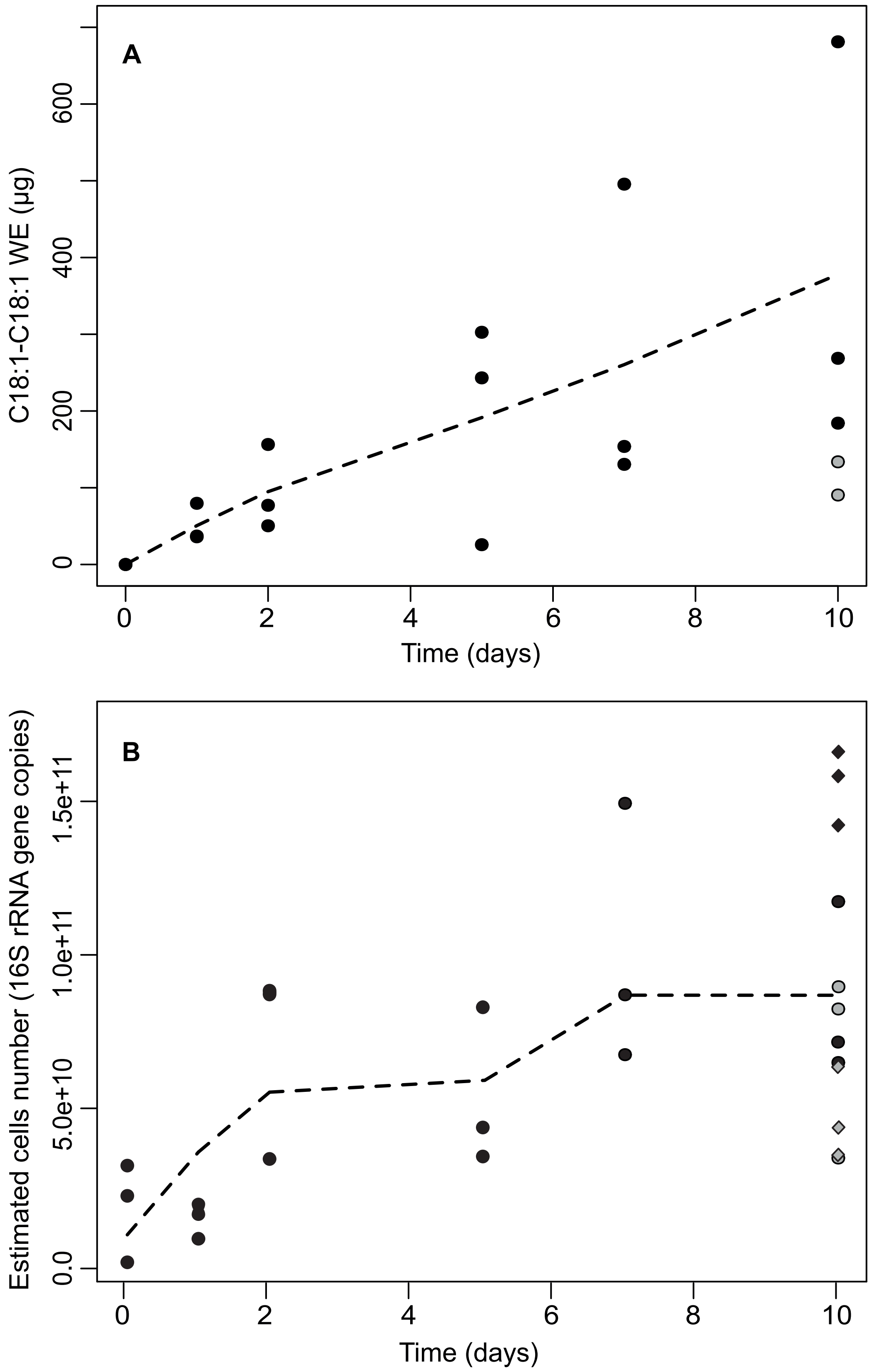

Figure S5: Amount of oleyl-oleate ($C_{18:1}$-$C_{18:1}$ WE) (**A**) and approximated cell number (number of 16S rRNA gene copies) (**B**) in cultures of N. pharaonis incubated in N-limited (black) and N-rich (grey) media supplemented with $C_{18:1}$ LCFA (circles) or soluble substrates (diamonds). Each data point corresponds to an individual culture. The dotted lines illustrate the loess regression of WE content and cell number during growth on $C_{18:1}$ LCFA under N-limited conditions.

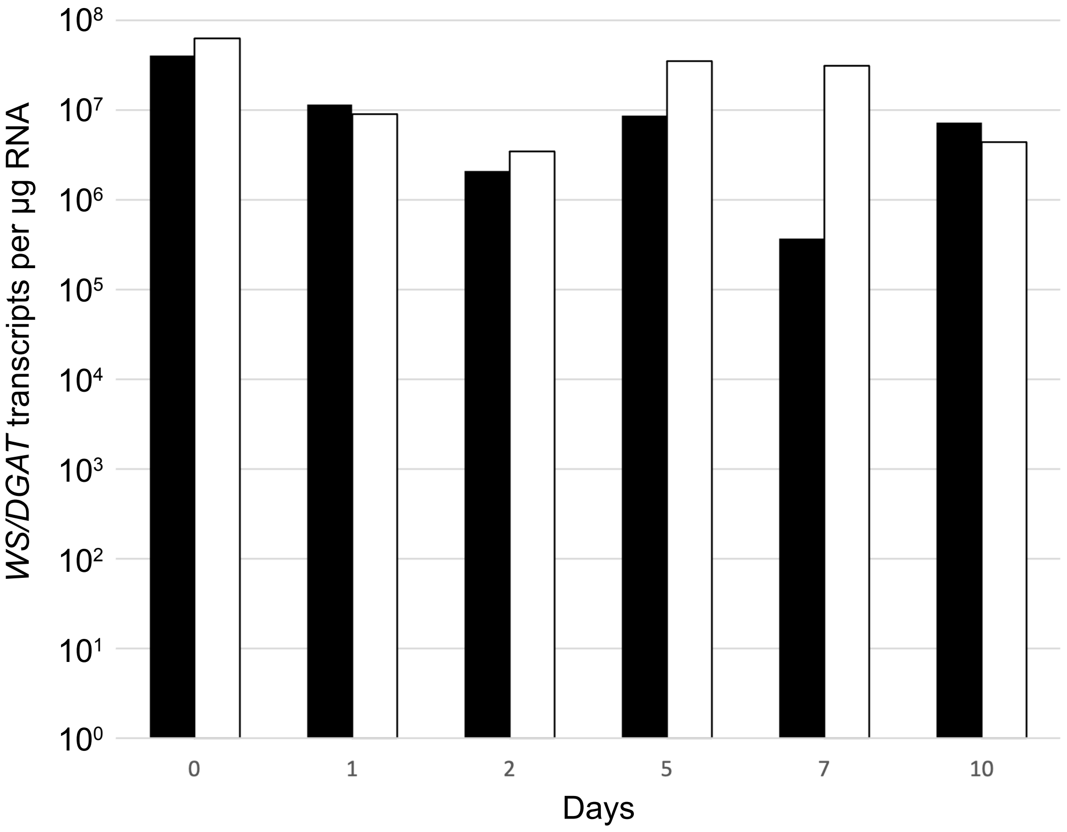

Figure S6: Expression of *N. pharaonis* WS/DGAT in N-limited medium supplemented with oleic acid (black) and soluble substrates (pyruvate and acetate, white). The number of transcripts was estimated by qPCR using specific primers. Each bar corresponds to an individual culture.

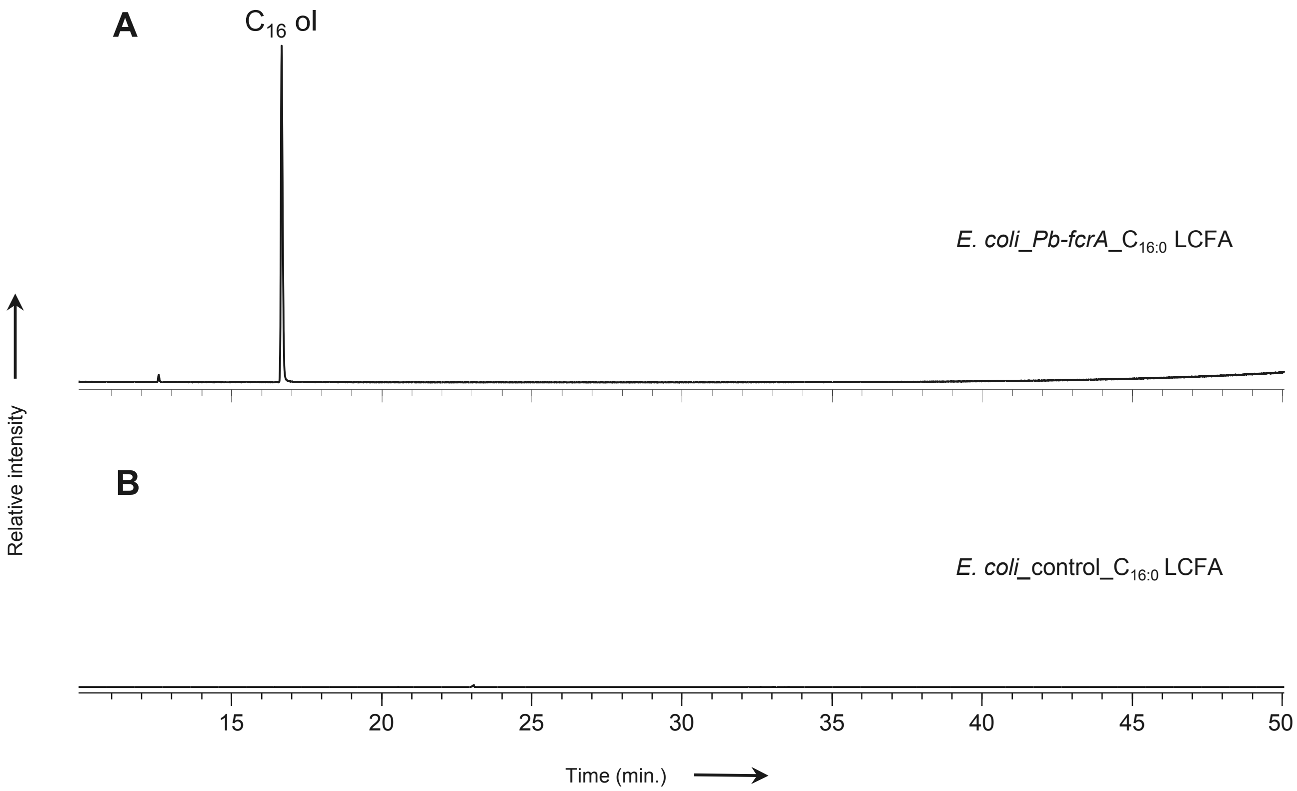

Figure S7: Mass chromatograms (m/z 75+103+299) of the alcohol fraction isolated from *E. coli* grown on $C_{16:0}$ LCFA and expressing (**A**) *Pb-fcrA* without *Ab-WS/DGAT*, (**B**) neither *Pb-fcrA* nor *Ab-WS/DGAT* (empty vector).

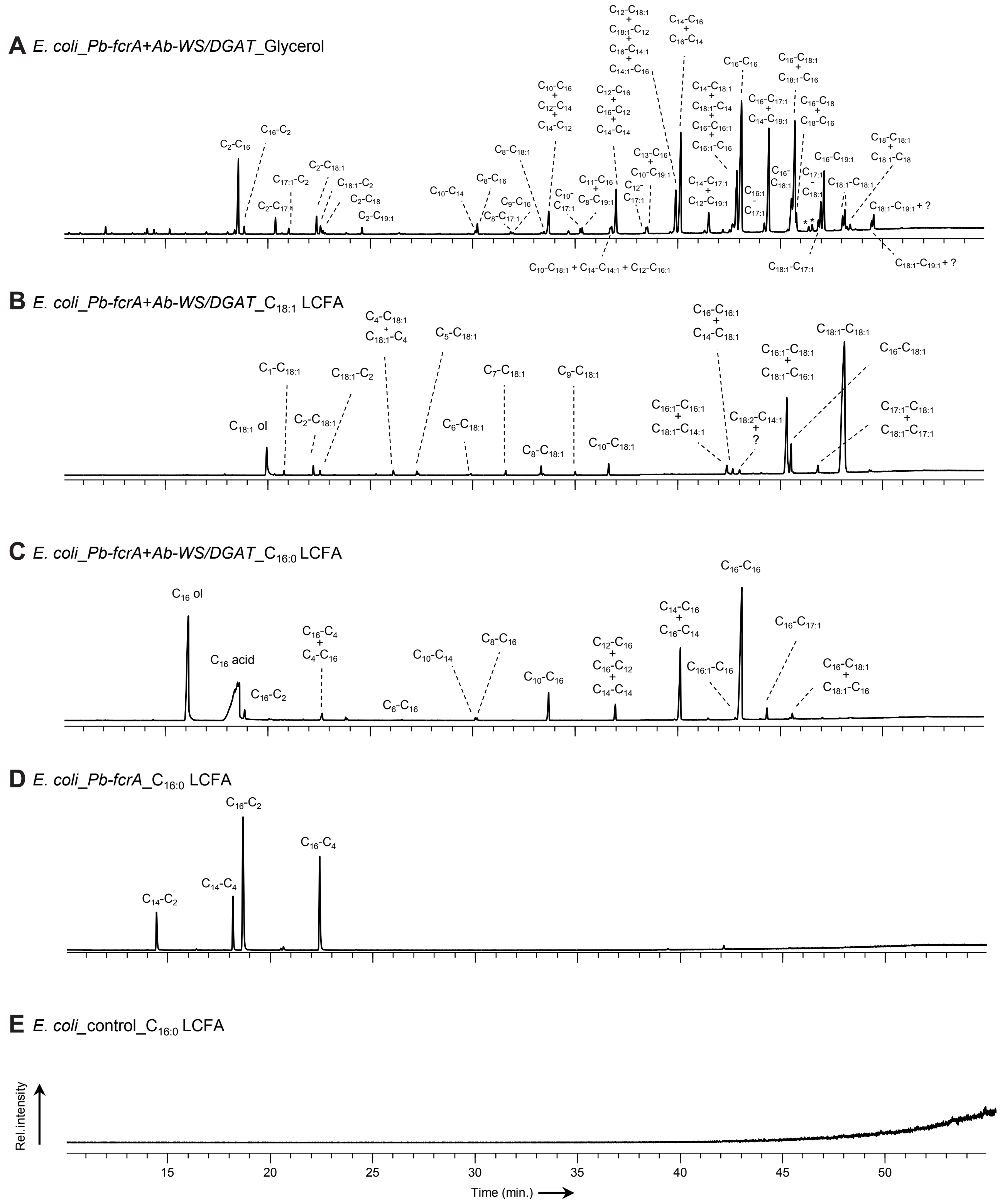

Figure S8: GC-MS total ion chromatograms (TIC) of the wax ester (WE) fraction isolated from *E. coli* (**A**) grown on glycerol and expressing *Pb-fcrA* and *Ab-WS/DGAT*; (**B**) grown on $C_{16:0}$ LCFA and expressing *Pb-fcrA* and *Ab-WS/DGAT*; (**C**) grown on $C_{18:1}$ LCFA and expressing *Pb-fcrA* and *Ab-WS/DGAT*; (**D**) grown on $C_{16:0}$ LCFA and expressing *Pb-fcrA* without *Ab-WS/DGAT*; (**E**) grown on $C_{16:0}$ LCFA and expressing neither Pb-fcrA nor Ab-WS/DGAT (empty vector). WE are annotated as $C_{x:x'}$-$C_{y:y'}$ with x carbon atoms and x' double bond in the alkyl chain and y carbon atoms and y' double bond in the acyl chain. When x' or y' are not indicated, the carbon chain is saturated. The presence of LCFA substrate and/or the corresponding alcohol in the WE fractions during growth on $C_{16:0}$ LCFA and $C_{18:1}$ LCFA is due to overloading of the silica gel column used for lipid separation. The presence of WE when Pb-fcrA is expressed in the absence of *Ab-WS/DGAT* (**D**) suggests WE production by an unknown enzyme in *E. coli*, possibly an unspecific reaction due to the accumulation of fatty-alcohol produced by *Pb-fcrA*. A contamination can be ruled out as the WE profile detected is clearly different from other profiles (**A**-**C**).

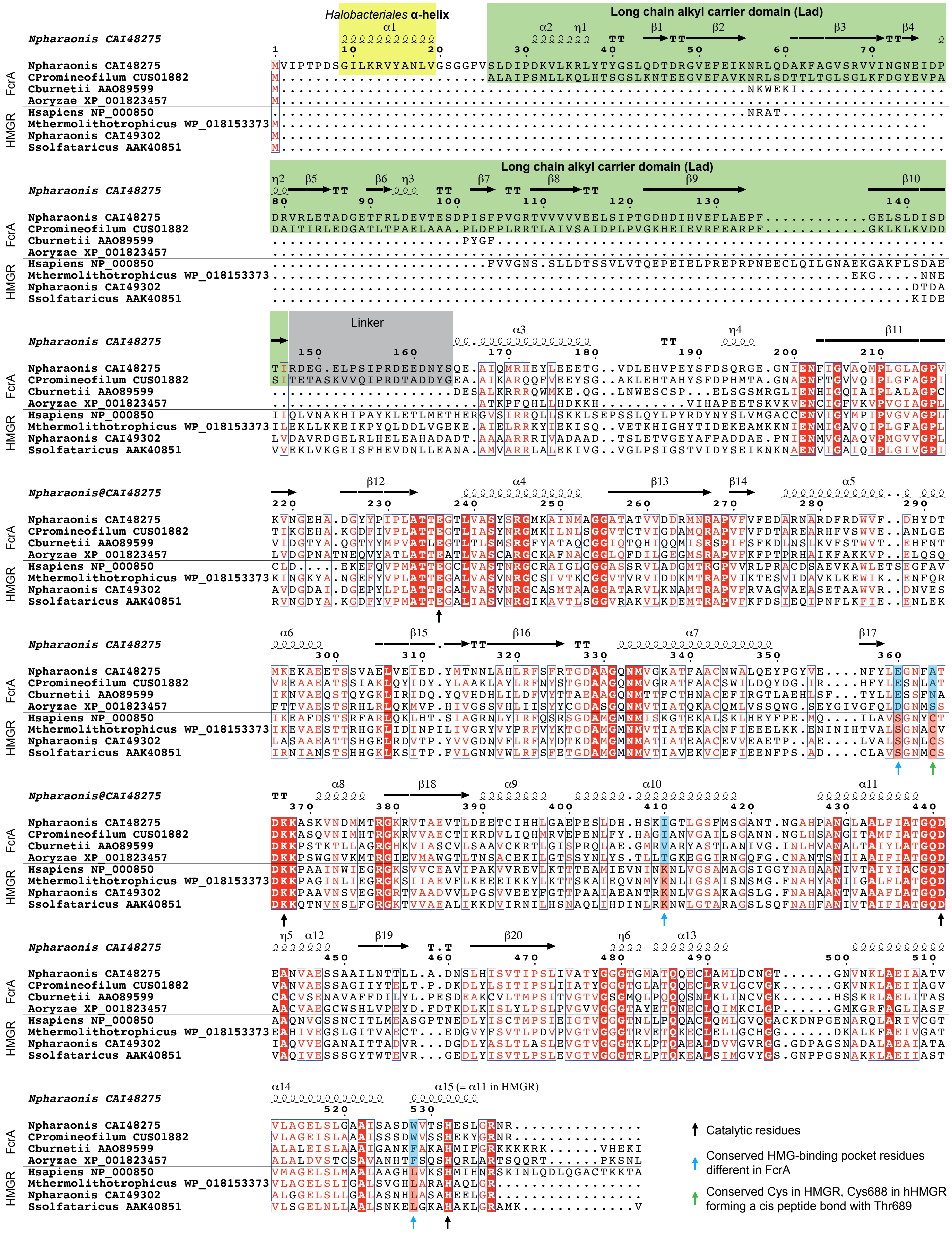

Figure S9: Sequence conservation between FcrA (secondary HMGR in *Halobacteriales*) and HMGR, generated with the Espript server. Black arrows indicate conserved catalytic amino-acids, blue arrows indicate two positions where FcrA and HMGR have different conserved amino-acids, which may play a role in substrate selection between HMG-CoA and acyl-CoA.

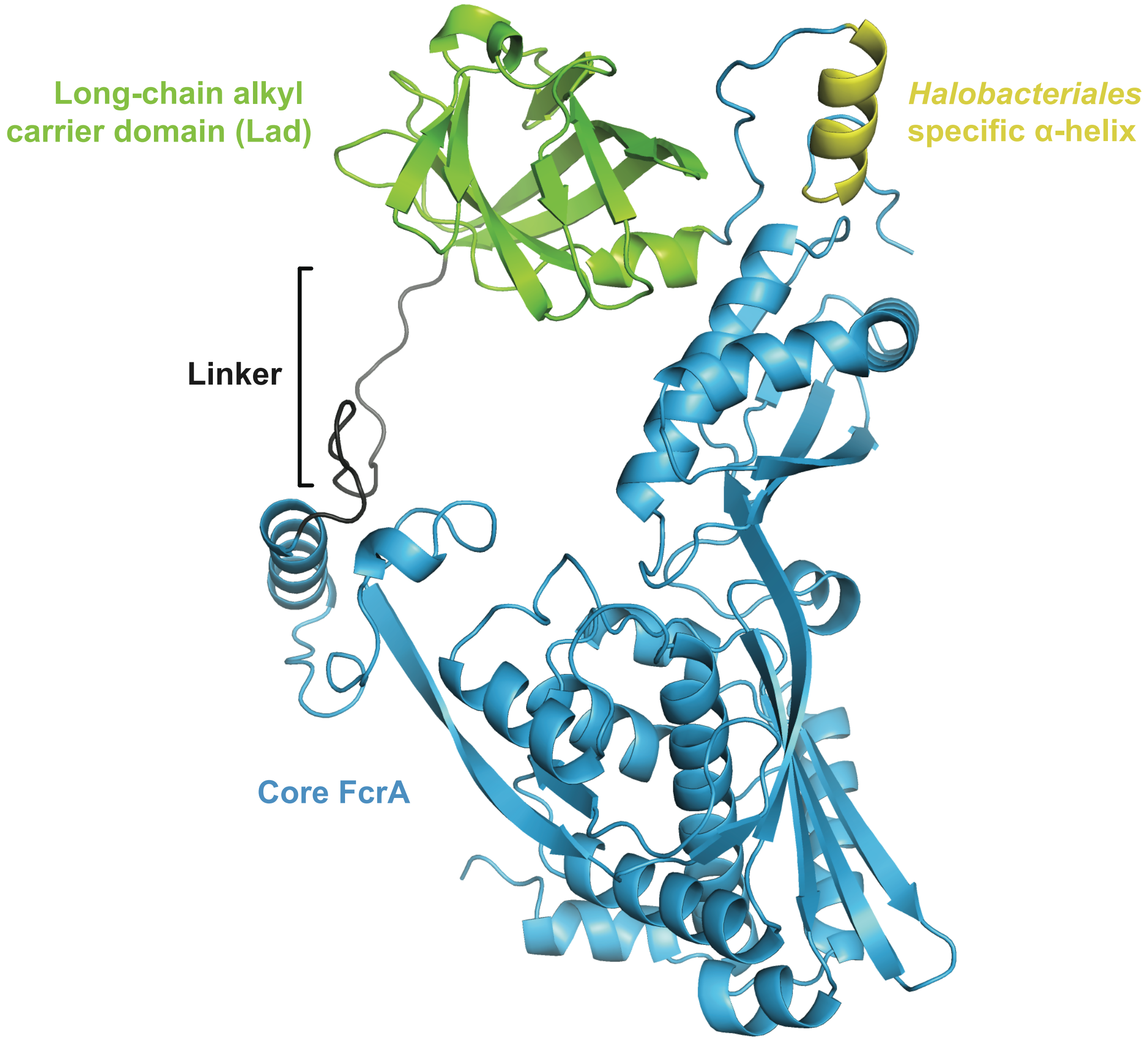

Figure S10: Structural model of the *N. pharaonis* FcrA monomer highlighting specific features.

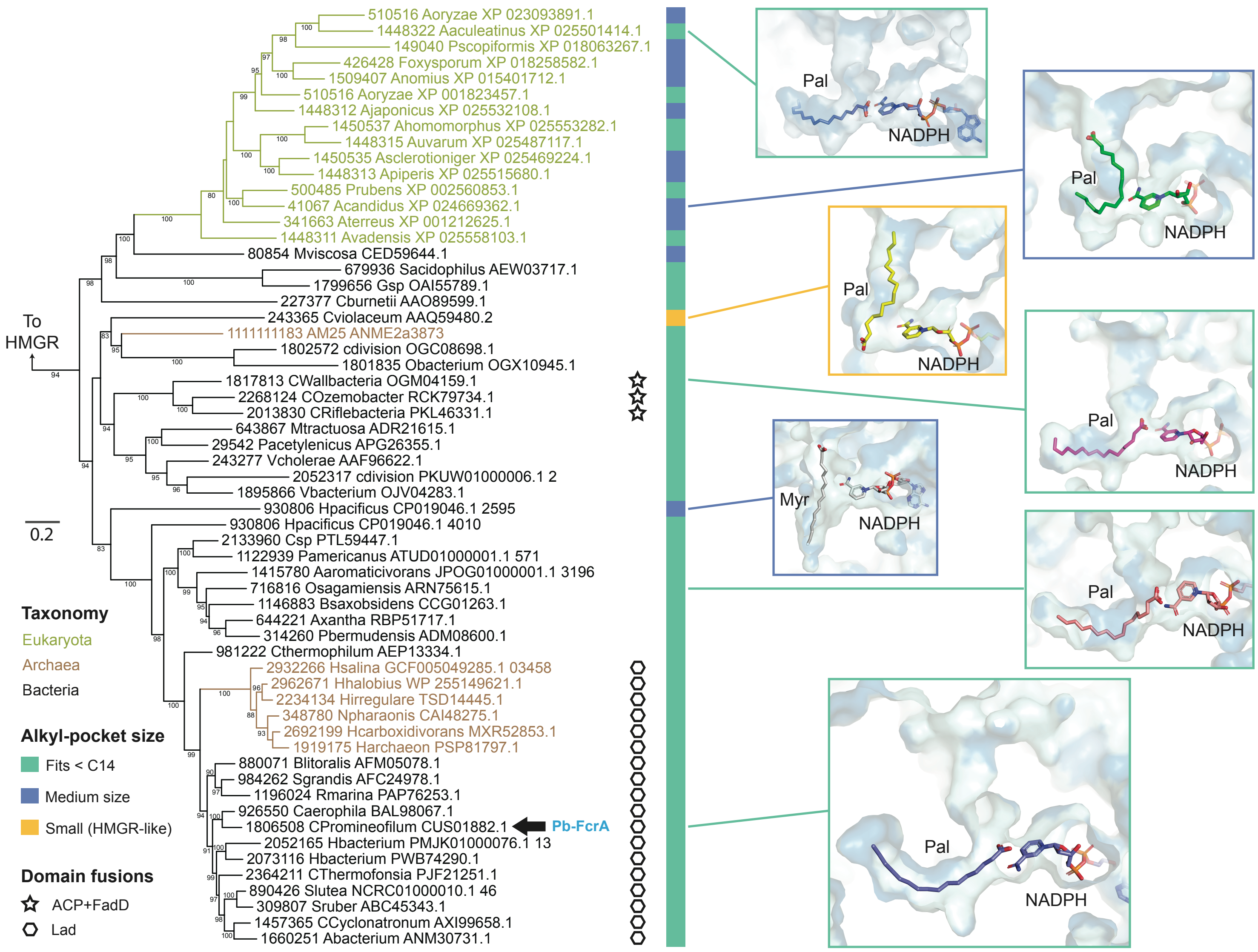

Figure S11: Distribution of the potential alkyl-binding pocket on the FcrA tree. The tree corresponds to the FcrA part of Fig. 4D. Symbols in front of the structure indicate the fusion of two types of additional N-terminal domains (star, FadD+ACP; hexagon, Lad). Examples of substrate-binding pockets of various size are shown on the right. Myristic (Myr) and palmitic (Pal) acids were used as an indicator of the position of the alkyl chain of myristoyl/palmitoyl-CoA and were positioned by AlphaFold 3, together with NADPH.

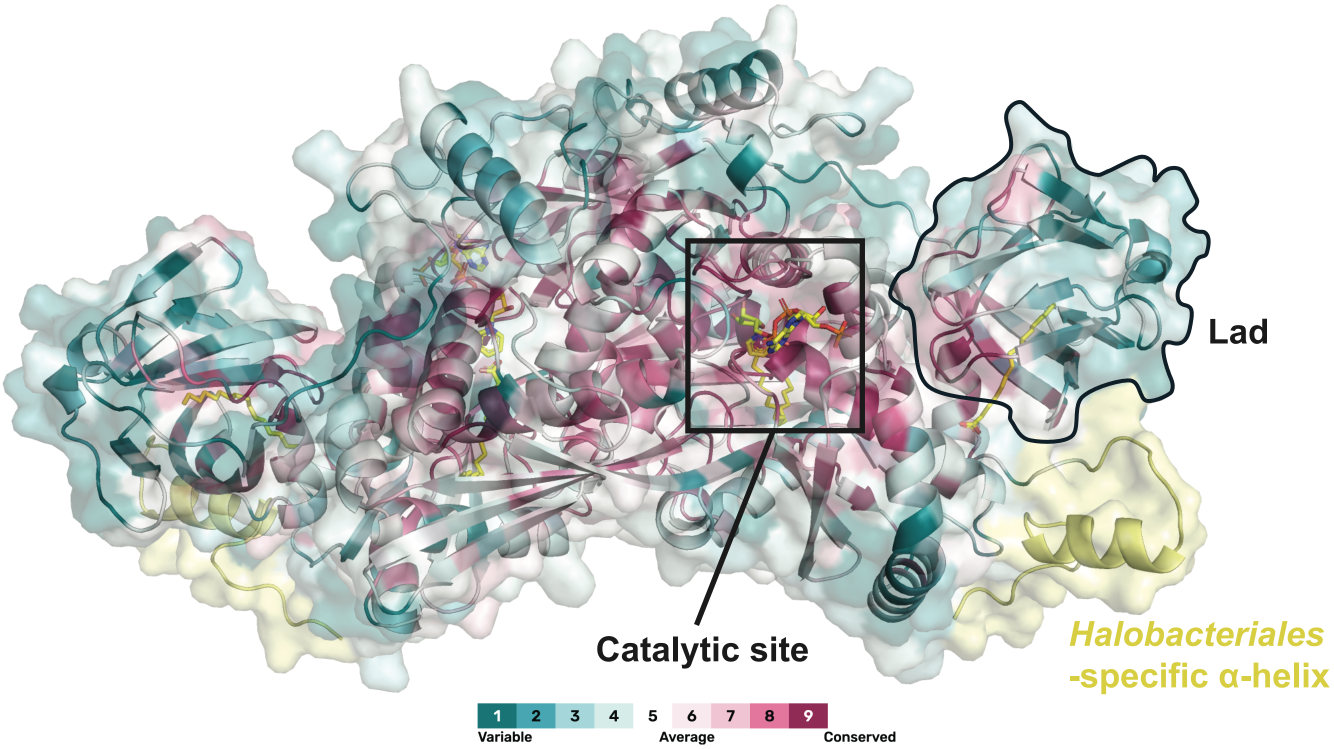

Figure S12: Conservation of residues mapped onto the structural model of an FcrA homodimer from *N. pharaonis*. The figure was generated using Consurf server and the FcrA sequences in the alignment used for Fig. 4D. One of the two Lad and the catalytic site are indicated, as well as an N-terminal helix only present in *Halobacteriales* (yellow, not enough data to estimate conservation).

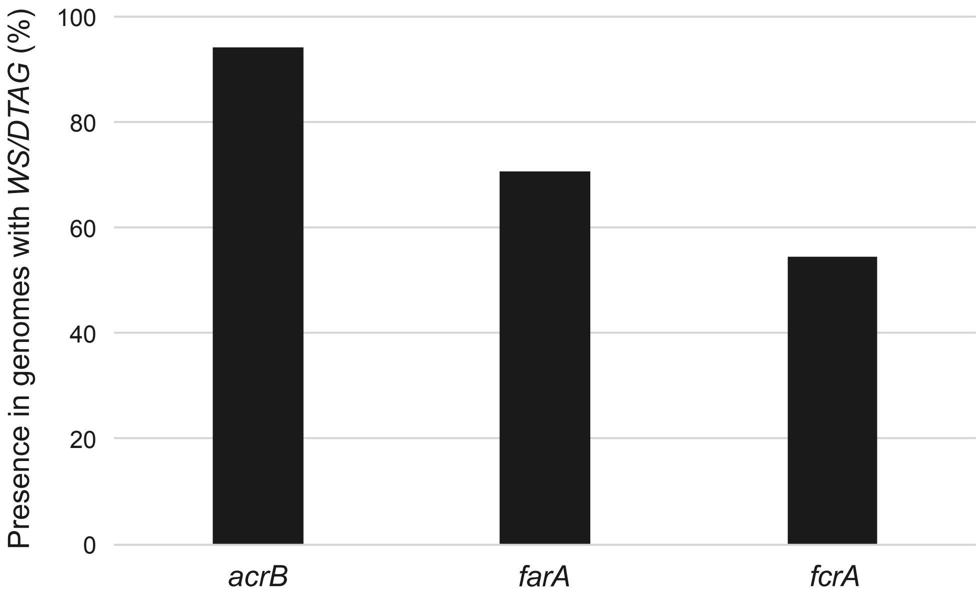

Figure S13: Percentage of fatty acyl-CoA reductase genes (*acrB*, *farA* and *fcrA*) present in genomes coding for WS/DGAT. The *acrB* gene is almost exclusively present in genomes also coding for WS/DGAT and is thus likely specifically involved in WE synthesis. In comparison, *farA* and *fcrA* are regularly present in genomes without *WS/DGAT*, indicating their involvement in pathways other than WE synthesis. Based on the genomic catalog of Earth's microbiomes [8].

SUPPLEMENTARY TABLES

Supplementary Table 1: Primers used in this study. The part of the primer that does not hybridize with the template DNA is indicated by bold characters.

Primer	Sequence 5' – 3'	Description
931F	AGGAATTGGCGGGGGAGCA	Forward primer - archaeal 16S rRNA gene
1100R	YGGGTCTCGCTCGTTRCC	Reverse primer - archaeal 16S rRNA gene
F_WS/DGAT_DSM2160	TTCCTGATGTACGCGCTGTT	Forward primer – RT-PCR of *WS/DGAT*
JW326	GCGACTCCTGCATTAGGAAG	Sequencing primer
JW327	CCCCAAGGGGTTATGCTAGT	Sequencing primer
JW381	**TTTAAGAAGGAGATATACAT**ATGGCTCTGGCTATCCCATC	Forward primer - FcrA for cloning in Nde of pET22b
JW382	**TGGTGGTGGTGGTGCTCGAG**GTTCGCCGTTTTGATGTC	Reverse primer - AcrA for cloning in XhoI of pET22b
JW383	GCGGAGTGCACCATCAAACG	Sequencing primer
JW384	CACGCATGTGCTGGATCAGC	Sequencing primer
JW385	GCACCACGGCGGAACGATGG	Sequencing primer
JW386	TGCTGGCATGCGTCTGATCG	Sequencing primer
JW397	**ACCGCGAACAGATTGGTGGC**ATGGCTCTGGCTATCCCATC	Forward primer - cloning FcrA into pT7-Sumo
JW398	**CTTTGTTAGCAGCCGGATCT**TTAACGGTTGCGGCCGTATTTTTC	Reverse primer - cloning FcrA into pT7-Sumo
pT7-F	AGATCCGGCTGCTAACAAAGC	Reverse - linearizing pT7-SUMO
pT7-R	GCCACCAATCTGTTCGCGG	Forward - linearizing pT7-SUMO
R_WS/DGAT_DSM2160	ATGTCGGTTATCTCGCGTCC	Reverse primer – RT-PCR of *WS/DGAT*

Supplementary Table 2: Plasmids used in this study.

Plasmid	Description	Reference or Source
pET22b	*E. coli* expression vector with a C-terminal his tag	Merck
pJW105	FcrA and WS/DGAT cloned as an artificial operon into pET22b digested NdeI/XhoI	This work
pJW111	pET22b containing only FcrA (no tag, obtained by EcoRI/XhoI excision from pJW105)	This work
pJW113	pT7-SUMO with FcrA cloned	This work
pJW114	pET22b containing only WS/DGAT	This work
pT7-Sumo	*E. coli* expression vector adding a N-terminal his tag followed by the SUMO sequence	[9]

Supplementary Table 3: Distribution of protein families most correlated with WS/DGAT in *Halobacteriales*. The Pearson correlation coefficient between the distribution pattern of a protein family and WS/DGAT is shown at the bottom. The first protein family (HaloFam45_004490) corresponds to WS/DGAT and the second (HaloFam45_004497) to fatty acyl-CoA reductase (FcrA). Information about Natronomonas pharaonis is in bold.

				Protein families																			
Assembly	Family	Species	WS/DGAT	HaloFam45_004490 CAI48224.1	HaloFam45_004497 CAI48275.1	HaloFam45_005971 TSD13365.1	HaloFam45_004513 CAI48449.2	HaloFam45_004654 CAI49560.1	HaloFam45_004564 CAI48936.1	HaloFam45_004548 CAI48779.1	HaloFam45_004653 CAI49559.1	HaloFam45_004699 CAI49784.1	HaloFam45_006477 TSD14397.1	HaloFam45_004650 CAI49546.1	HaloFam45_004542 CAI48722.1	HaloFam45_004809 CAI50732.1	HaloFam45_004649 CAI49543.1	HaloFam45_006120 TSD13807.1	HaloFam45_006098 TSD13681.1	HaloFam45_005560 TSD13896.1	HaloFam45_004766 CAI50305.1	HaloFam45_004706 CAI49821.1	HaloFam45_004648 CAI49488.1
GCF024298885	Haloarculaceae	Haloglomus halophilus ZY41	Yes	2	1	1	1	1	1	3	1	3	1	4	1	3	1	2	1	1	1	2	12
GCF024300825	Haloarculaceae	Natronomonas gomsonensis KCTC 4088	Yes	1	1	1	2	2	1	3	1	2	1	2	1	2	1	1	1	1	1	2	8
GCA003020985	Haloarculaceae	Halobacteriales archaeon QS_4_70_19	Yes	1	1	1	1	1	1	2	1	3	1	1	1	4	1	2	1	1	1	1	8
GCF024298905	Haloarculaceae	Natronomonas marina ZY43	Yes	1	1	1	1	1	1	3	1	2	1	1	1	2	1	1	1	1	1	3	7
GCA013391085	Haloarculaceae	Natronomonas halophila C90	Yes	1	1	1	1	2	1	2	2	2	1	2	1	2	1	1	1	1	1	3	7
GCF024362405	Haloarculaceae	Salinirubellus sp. BND22	Yes	1	1	1	1	1	1	2	1	2	1	2	1	2	1	1	1	1	1	3	9
GCF007421925	Haloarculaceae	Haloglomus irregulare	Yes	1	1	1	1	1	1	1	1	2	1	2	1	2	1	1	1	1	1	1	6
GCF024298865	Haloarculaceae	Halorarius halobius ZY10	Yes	1	1	0	1	1	1	1	1	2	1	3	1	2	1	1	1	1	1	2	8
GCA003020965	Haloarculaceae	Halobacteriales archaeon QS_4_69_225	Yes	1	1	1	0	1	1	1	1	1	0	1	1	3	1	1	1	1	1	0	4
**GCA000026045**	**Haloarculaceae**	**Natronomonas pharaonis DSM 2160**	**Yes**	**1**	**1**	**0**	**1**	**1**	**1**	**1**	**1**	**1**	**0**	**1**	**1**	**1**	**1**	**0**	**0**	**0**	**1**	**1**	**2**
GCA003551945	Haloarculaceae	Halovenus sp. B1Sed10_163	Yes	1	1	1	1	1	0	1	1	1	0	0	0	0	0	0	0	0	0	1	1
GCF009831575	Haloarculaceae	Halovenus carboxidivorans WSH3	Yes	1	1	1	1	1	0	1	1	1	0	0	0	0	0	0	0	0	0	1	0
GCF000416085	Haloarculaceae	Halovenus archaeon J07HX64	Yes	1	0	0	1	1	0	1	1	1	0	0	0	0	0	0	0	1	0	1	1
GCF013391105	Haloarculaceae	Natronomonas salina	No	0	0	0	4	1	1	2	1	2	0	1	1	2	1	1	1	1	1	2	9
GCA003021175	Haloarculaceae	Haloarculaceae archaeon QH_7_66_36	No	0	0	0	0	0	0	0	0	0	0	0	0	0	0	0	0	0	0	0	0
GCF000591055	Haloarculaceae	Natronomonas moolapensis 8.8.11	No	0	0	0	0	0	0	0	0	0	0	0	1	0	1	0	0	0	0	0	0
GCA009791395	Haloarculaceae	Halomarina oriensis	No	0	0	0	0	1	0	1	1	1	0	0	0	0	0	0	0	0	1	1	0
GCF005049285	Haloarculaceae	Halorientalis archaeon NEN8	No	0	1	0	1	1	0	3	1	1	0	1	0	0	0	0	0	0	0	2	5
GCF009831455	Haloarculaceae	Salinirussus salinus	No	0	0	0	0	1	0	0	1	1	0	0	0	0	0	0	0	0	0	1	0
GCA000023965	Haloarculaceae	Halomicrobium mukohataei DSM 12286	No	0	0	0	0	0	0	0	0	0	0	0	0	0	0	0	0	0	0	0	0
GCA016065055	Haloarculaceae	Halosimplex litoreum	No	0	0	0	0	0	0	0	0	0	0	0	0	0	0	0	0	0	0	0	0
GCF005310945	Haloarculaceae	Haloarcula marismortui ATCC 43049	No	0	0	0	0	0	0	0	0	0	0	0	0	0	0	0	0	0	0	0	0
GCA003021045	Halococcaceae	Halococcus archaeon QS_4_69_34	No	0	0	0	1	1	0	1	1	2	0	0	0	0	0	0	0	1	0	1	2
GCF002844195	Haloferacaceae	Halegenticoccus soli	No	0	0	0	0	0	0	0	0	0	0	0	0	1	0	0	0	0	0	0	2
GCA000025625	Natrialbaceae	Natrialba magadii ATCC 43099	No	0	0	0	0	0	0	0	0	0	0	0	0	0	0	0	0	0	0	0	0
GCA900103505	Natrialbaceae	Haloarchaeobius iranensis	No	0	0	0	0	0	0	1	0	0	0	0	0	0	0	0	0	0	0	0	0
GCA003021085	PXRE01	Halobacteriales archaeon QS_1_68_20	No	0	0	0	0	0	0	0	1	1	0	0	0	0	0	0	0	0	0	0	0
GCF020700235	Haladaptataceae	Haladaptatus pallidirubidus	No	0	0	0	0	0	0	0	0	0	0	0	0	0	0	0	0	0	0	0	1
GCA017094485	Natronoarchaeaceae	Natronoarchaeum archaeon AArc-S	No	0	0	0	0	0	0	0	0	0	0	0	0	0	0	0	0	0	0	0	0
GCF000403645	Salinarchaeaceae	Salinarchaeum sp. Harcht-Bsk1	No	0	0	0	0	0	0	0	0	0	0	0	0	0	0	0	0	0	0	0	0
GCA000196895	Halalkalicoccaceae	Halalkalicoccus jeotgali B3	No	0	0	0	0	0	0	0	0	0	0	0	0	0	0	0	0	0	0	0	0
GCF009900715	Halococcaceae	Halococcus salsus	No	0	0	0	0	0	0	0	0	0	0	0	0	0	0	0	0	0	0	0	0
GCF003605635	Haloferacaceae	Halonotius pteroides	No	0	0	0	0	0	0	0	0	0	0	0	0	0	0	0	0	0	0	0	0
GCA000328525	Natrialbaceae	Halovivax ruber XH-70	No	0	0	0	0	0	0	0	0	0	0	0	0	0	0	0	0	0	0	0	0
GCF004087835	Haladaptataceae	Halorussus sp. RC-68	No	0	0	0	0	0	0	0	0	0	0	0	0	0	0	0	0	0	0	0	0
GCA000023945	Haloarculaceae	Halorhabdus utahensis DSM 12940	No	0	0	0	0	0	0	0	0	0	0	0	0	0	0	0	0	0	0	0	0
GCA000006805	Halobacteriaceae	Halobacterium salinarum NRC-1	No	0	0	0	0	0	0	0	0	0	0	0	0	0	0	0	0	0	0	0	0
GCF020614395	Halobacteriaceae	Salarchaeum japonicum	No	0	0	0	0	0	0	0	0	0	0	0	0	0	0	0	0	0	0	0	0
GCA003021725	Halococcaceae	Halococcus archaeon QH_8_64_26	No	0	0	0	0	0	0	0	0	0	0	0	0	0	0	0	0	0	0	0	0
GCA000022205	Haloferacaceae	Halorubrum lacusprofundi ATCC 49239	No	0	0	0	0	0	0	0	0	0	0	0	0	0	0	0	0	0	0	0	0
GCA000495475	Haloferacaceae	Halobonum tyrrellensis G22	No	0	0	0	0	0	0	0	0	0	0	0	0	0	0	0	0	0	0	0	0
GCF000009185	Haloferacaceae	Haloquadratum walsbyi DSM 16790	No	0	0	0	0	0	0	0	0	0	0	0	0	0	0	0	0	0	0	0	0
GCF005954745	QS-9-68-17	Halostella pelagica	No	0	0	0	0	0	0	0	0	0	0	0	0	0	0	0	0	0	0	0	0
GCF001886955	Halobacteriaceae	Halodesulfurarchaeum formicicum	No	0	0	0	0	0	0	0	0	0	0	0	0	0	0	0	0	0	0	0	0
		Pearson Correlation Coefficient		1	0.89	0.84	0.8	0.78	0.78	0.78	0.74	0.74	0.73	0.72	0.72	0.72	0.72	0.72	0.72	0.72	0.72	0.71	0.71

Supplementary Table 4: *Escherichia coli* strains used in this study.

Strain	Genotype	Reference or Source
DH5α	*F- endA1 glnV44 thi-1 recA1 relA1 gyrA96 deoR nupG purB20* φ80d*lacZ*ΔM15 Δ(*lacZYA-argF*)U169, hsdR17($r_K^- m_K^+$), λ^-	Promega
BL21(DE3)	*F- ompT hsdSB(rB–mB–) gal dcm (DE3); host for protein production*	[10]
C43(DE3)	F – ompT hsdSB (rB- mB-) gal dcm (DE3)	Merck

References for supplementary methods and figures

1. Lee ZM-P, Bussema III C, Schmidt TM. rrn DB: documenting the number of rRNA and tRNA genes in bacteria and archaea. Nucleic Acids Res 2009; 37: D489–D493.

2. McDonald KL. Out with the old and in with the new: rapid specimen preparation procedures for electron microscopy of sectioned biological material. Protoplasma 2014; 251: 429–448.

3. Reynolds ES. The use of lead citrate at high pH as an electron-opaque stain in electron microscopy. J Cell Biol 1963; 17: 208.

4. Urbanová K, Vrkoslav V, Valterová I, Háková M, Cvacka J. Structural characterization of wax esters by electron ionization mass spectrometry. J Lipid Res 2012; 53: 204–213.

5. Green MR, Sambrook J. Molecular cloning. A Laboratory Manual 4th . 2012. Cold Spring Harbor Laboratory Press.

6. Untergasser A, Nijveen H, Rao X, Bisseling T, Geurts R, Leunissen JAM. Primer3Plus, an enhanced web interface to Primer3. Nucleic Acids Res 2007; 35: W71–W74.

7. Inoue H, Nojima H, Okayama H. High efficiency transformation of Escherichia coli with plasmids. Gene 1990; 96: 23–28.

8. Nayfach S, Roux S, Seshadri R, Udwary D, Varghese N, Schulz F, et al. A genomic catalog of Earth's microbiomes. Nat Biotechnol 2021; 39: 499–509.

9. Studier FW, Moffatt BA, Use of bacteriophage T7 RNA polymerase to direct selective high-level expression of cloned genes. J Mol Biol1986; 189: 113-130.

10. Pende N, et al., SepF is the FtsZ anchor in archaea, with features of an ancestral cell division system. Nat. Commun 2021; 12: 3214.
